# Expedited transfer to a cardiac arrest centre for non-ST-elevation out-of-hospital cardiac arrest (ARREST): a UK prospective, multicentre, parallel, randomised clinical trial

**DOI:** 10.1016/S0140-6736(23)01351-X

**Published:** 2023-10-14

**Authors:** Tiffany Patterson, Gavin D Perkins, Alexander Perkins, Tim Clayton, Richard Evans, Matthew Dodd, Steven Robertson, Karen Wilson, Adam Mellett-Smith, Rachael T Fothergill, Paul McCrone, Miles Dalby, Philip MacCarthy, Sam Firoozi, Iqbal Malik, Roby Rakhit, Ajay Jain, Jerry P Nolan, Simon R Redwood, ARREST trial collaborators, Divaka Perera, Brian Clapp, Bernard Prendergast, Antonis Pavlidis, Andrew Wragg, Jonathan Byrne, Nigel Stephens, Gareth Rosser, Darryl Wood, Robert Bell, Arvinder Kurbaan, Muhiddin Ozkor, Anthony Lampard, Desiree Papadopoulos, Johanna Hughes, Valentina Pendolino, Joanna Shaw, Clara Bannister, Amy Long, Justin Kearney, Gabriel Palti, Joanne Ritches-Price, Mark Whitbread, Dawn Adamson, Lucy Blows, Martin Brown, Garth Lane, Michael Connor, Keith Muir, Douglas Chamberlain, Tim Morris, Matthew Kwok, Megan Knight, Lauren Jerome, Sukhjinder Nijjer, Rita Das, Therese Sidney, Richard Bogle, Patrick Roberts, Ian Webb, Oliver Spencer, Edward Petzer, Masood Khan, Maciej Marciniak, Mark De Belder, Rod Stables, Nick Curzen, Mamas Mamas

**Affiliations:** a Cardiovascular Department, Guy's and St Thomas' NHS Foundation Trust, London, UK; b Cardiovascular Department, Faculty of Life Sciences and Medicine, King's College London, London, UK; c Clinical Trials Unit, Warwick Medical School, University of Warwick, Coventry, UK; d London School of Hygiene & Tropical Medicine Clinical Trials Unit, London, UK; e Clinical Audit and Research Unit, London Ambulance Service, London, UK; f Faculty of Health, Social Care and Education, Kingston University and St George's, University of London, London, UK; g Institute for Lifecourse Development, University of Greenwich, London, UK; h Department of Cardiology, Brompton and Harefield NHS Foundation Trust, London, UK; i Department of Cardiology, King's College Hospital, London, UK; j Department of Cardiology, St Georges Hospital, London, UK; k Department of Cardiology, Imperial College Healthcare NHS Trust, London, UK; l Department of Cardiology, Royal Free Hospital Foundation Trust, London, UK; m Department of Cardiology, Barts Heart Centre, London, UK; n Department of Anaesthesia, Royal United Hospital, Bath, UK

## Abstract

**Background:**

The International Liaison Committee on Resuscitation has called for a randomised trial of delivery to a cardiac arrest centre. We aimed to assess whether expedited delivery to a cardiac arrest centre compared with current standard of care following resuscitated cardiac arrest reduces deaths.

**Methods:**

ARREST is a prospective, parallel, multicentre, open-label, randomised superiority trial. Patients (aged ≥18 years) with return of spontaneous circulation following out-of-hospital cardiac arrest without ST elevation were randomly assigned (1:1) at the scene of their cardiac arrest by London Ambulance Service staff using a secure online randomisation system to expedited delivery to the cardiac catheter laboratory at one of seven cardiac arrest centres or standard of care with delivery to the geographically closest emergency department at one of 32 hospitals in London, UK. Masking of the ambulance staff who delivered the interventions and those reporting treatment outcomes in hospital was not possible. The primary outcome was all-cause mortality at 30 days, analysed in the intention-to-treat (ITT) population excluding those with unknown mortality status. Safety outcomes were analysed in the ITT population. The trial was prospectively registered with the International Standard Randomised Controlled Trials Registry, 96585404.

**Findings:**

Between Jan 15, 2018, and Dec 1, 2022, 862 patients were enrolled, of whom 431 (50%) were randomly assigned to a cardiac arrest centre and 431 (50%) to standard care. 20 participants withdrew from the cardiac arrest centre group and 19 from the standard care group, due to lack of consent or unknown mortality status, leaving 411 participants in the cardiac arrest centre group and 412 in the standard care group for the primary analysis. Of 822 participants for whom data were available, 560 (68%) were male and 262 (32%) were female. The primary endpoint of 30-day mortality occurred in 258 (63%) of 411 participants in the cardiac arrest centre group and in 258 (63%) of 412 in the standard care group (unadjusted risk ratio for survival 1·00, 95% CI 0·90–1·11; p=0·96). Eight (2%) of 414 patients in the cardiac arrest centre group and three (1%) of 413 in the standard care group had serious adverse events, none of which were deemed related to the trial intervention.

**Interpretation:**

In adult patients without ST elevation, transfer to a cardiac arrest centre following resuscitated cardiac arrest in the community did not reduce deaths.

**Funding:**

British Heart Foundation.

## Introduction

There are marked regional variations in survival following resuscitated out-of-hospital cardiac arrest (OHCA), which are attributable to resources, personnel, and infrastructure in addition to patient characteristics.^1^, ^2^, ^3^ Regionalisation of care improves outcomes in patients with time-critical illness by concentrating services within centres, increasing the number of patients treated and therefore the skills and experience of health-care providers within those centres.^4^ Implementing prehospital systems of care for OHCA management would work in a similar manner to networks for ST-elevation myocardial infarction, with ambulance staff providing prompt identification and delivery of patients to a designated cardiac arrest centre.^5^, ^6^ Post-arrest care with early interventions for ischaemia-reperfusion injury and treatment of the underlying cause has preferential outcomes.^7^ This care might be better delivered in a cardiac arrest centre; however, observational studies yield conflicting results due to confounding variables, including selection bias and heterogeneity of care.^8^ As a result, the International Liaison Committee on Resuscitation highlighted the need for a randomised trial. Having established feasibility through a pilot randomised trial, we did a prospective, randomised controlled study (the ARREST trial) to establish whether the delivery of patients to a cardiac arrest centre reduces deaths.^9^, ^10^

**Research in context**

**Evidence before this study**

We did a literature search of PubMed with the search terms “cardiac arrest centre” or “cardiac arrest center” within the title and abstract on July 28, 2016, before embarking on this study to determine whether any previous trials in this area had been conducted; further annual searches of the International Standard Randomised Controlled Trials Registry and ClinicalTrials.gov with the search terms “cardiac arrest centre” or “cardiac arrest center” confirmed that no similar trials were planned or had been previously conducted. Data from a large meta-analysis suggests that delivering patients after arrest to a cardiac arrest centre improves survival; however, these data are subject to bias. There is a strong drive internationally to create cardiac arrest networks on the basis of a potential survival benefit. However, creation of a cardiac arrest network will have huge logistical challenges and cost implications.

**Added value of this study**

The International Liaison Committee on Resuscitation has called for randomised trials of cardiac arrest centres. To our knowledge, ARREST is the first and only randomised trial of delivery to a cardiac arrest centre following resuscitated out-of-hospital cardiac arrest in the community and addresses an important unknown in post-resuscitation care.

**Implications of all the available evidence**

After cardiac arrest, the likelihood of survival did not increase despite the patients being delivered to a well resourced cardiac arrest centre with access to multiple facilities aimed at improving outcome. This finding is in contrast to the results from previous observational studies of cardiac arrest centres that showed a survival benefit. However, the results are consistent with the findings of trials examining post-resuscitation care including immediate angiography in the non-ST-elevation population and hypothermia, which did not show a survival benefit. We show that delivery of patients to the nearest emergency department after presumed cardiac arrest without ST elevation is a reasonable approach and has similar outcomes as delivery to a tertiary centre in the UK health-care setting.

## Methods

### Study design

The ARREST trial is an investigator-led, prospective, parallel, multicentre, open-label, randomised superiority trial in which expedited transfer to a cardiac arrest centre was compared with current standard of care, comprising patient transfer to the geographically closest emergency department following resuscitated OHCA.

The study was conducted by the London Ambulance Service National Health Service (NHS) Trust (the primary provider of prehospital emergency care across Greater London) and in all 35 centres (acute hospitals capable of receiving patients from the London Ambulance Service) in London, UK. The geographical spread of the 35 hospitals across London is shown in the appendix (p 11). Seven of these hospitals were cardiac arrest centres with emergency out-of-hours provision for interventional cardiology, cardiac surgery, and specialist intensive-care facilities, four of which also had emergency departments. Details of the accessible facilities in the cardiac and non-cardiac arrest centres are provided in the appendix (p 13). Cardiac arrest centres adhered to the previously published minimum criteria (appendix p 18).^10^

In January, 2014, the National Research Ethics Committee granted ethics approval for the pilot trial (REC 13/LO/1508) and approved the full trial with amendments in 2017. The trial protocol was developed by the project management group and is available online.^10^ The trial conformed with the Declaration of Helsinki, was prospectively registered with the International Standard Randomised Controlled Trials Registry (ISRCTN96585404) and is closed to accrual.

### Participants

Due to the emergency scenario and immediacy of the intervention, and to replicate standard practice, the eligibility assessment was performed by paramedics at the scene of the cardiac arrest without investigator oversight. Patients (aged ≥18 years) with return of spontaneous circulation following OHCA were deemed eligible for enrolment. Exclusion criteria were presumed non-cardiac cause, pregnancy, criteria for ST-elevation myocardial infarction on the post-resuscitation 12-lead electrocardiogram (ECG), or presence of a do-not-attempt-resuscitation order. Full details of the inclusion and exclusion criteria are provided in the appendix (p 24).

Data on sex were most frequently recorded before patients regained consciousness and were recorded on the case report form by research nurses or research paramedics based on the patient's NHS records; options provided were male and female, and a category of unknown was to be assigned if the data could not be obtained.

The need for prior informed consent was waived because of the urgency of the intervention.^11^ Written informed consent was taken from the patient once the initial emergency had passed if they had regained capacity or from a personal or professional consultee. The Confidentiality Advisory Group granted permission (17/CAG/0151) to access patient-identifiable data under specific circumstances (appendix p 25).

### Randomisation and masking

Randomisation was performed by Advanced Paramedic Practitioners at the dispatch desk using a secure online randomisation system. Following resuscitated OHCA, attending paramedics randomly assigned patients (1:1) using block permutation (sizes 4 and 6) without stratification to expedited transfer to a cardiac arrest centre or standard-of-care treatment (delivery to the closest emergency department). It was not deemed possible to mask clinicians, ambulance staff, or participants to the assigned group due to the radically different treatment groups. Randomisation was paused twice for 8 months (March, 2020, through to November, 2020, and January, 2021, through to August, 2021) during the height of the COVID-19 pandemic due to increased operational pressures on the NHS. Full details on the randomisation process are provided in the appendix (pp 26–27)

### Procedures

The London Ambulance Service emergency response to OHCA has been previously published (appendix pp 26–27).^12^ London Ambulance Service resuscitation protocols follow European^13^ and international guidelines. Following resuscitated OHCA**,** patients in the cardiac arrest centre group were managed following the cardiac arrest pathway (24 h a day, 7 days per week), whereby the receiving centre was pre-alerted to the imminent arrival of a patient resuscitated from cardiac arrest with strategic delivery to the catheter laboratory.^9^, ^10^ Following delivery to the cardiac arrest centre, patients were assessed by the on-call team (including cardiologist and intensivist teams). There is a known discordance between the presumed cardiac cause assessed before the hospital visit and the definitive cause established in hospital or post mortem. Due to this heterogeneity of possible diagnoses, we did not dictate in-hospital management, which was left to physician discretion and expertise.^14^ Cardiac arrest centres were fully equipped and had access to guideline-directed therapies, including tracheal intubation and ventilation, haemodynamic support and monitoring, assessment of the underlying cause of arrest with on-site diagnostics, immediate reperfusion or mechanical support devices if necessary, temperature control, and appropriate neuroprognostication (appendix pp 13–17). Patients in the standard care group were transported to the geographically closest emergency department. Once the patients had been delivered to the assigned centre, they were considered as having completed the allocated trial assignment. Both groups were required to have delayed neuroprognostication after 72 h and before withdrawal of life-sustaining treatment.^15^ The final participant completed 3 months of follow-up in February, 2023.

The London School of Hygiene & Tropical Medicine Clinical Trials Unit coordinated and managed all aspects of trial conduct. A complete list of individual sites and investigators, the project management group, trial steering committee membership (responsible for trial oversight), and data and safety monitoring committee membership is provided in the protocol^10^ and appendix (pp 3–7).

### Outcomes

The primary endpoint was all-cause mortality at 30 days, which was assessed by the clinical teams, research nurses, or research paramedics (appendix pp 22–23). Secondary endpoints comprised mortality at 3 months, neurological (functional) outcomes at discharge and 3 months, and EQ-5D-5L at discharge. Data collection for 6-month and 12-month mortality is incomplete and will be reported separately with health-care costs and cost-effectiveness. Survival with favourable neurological outcome was defined as having a cerebral performance category score of 2 or less or a modified Rankin Scale (mRS) score of 3 or less.^16^ Serious unexpected adverse events were classed as any untoward medical occurrence or effect that: resulted in death, was life-threatening, required hospitalisation, prolonged the existing inpatient's hospitalisation, or resulted in persistent or substantial disability or incapacity. Serious expected adverse events (as a consequence of cardiac arrest) that were reported on the case report form but did not require a separate adverse event report were: death, myocardial infarction, stroke, neurological complications, and multi-organ failure. Expected adverse events that did not require separate adverse event reporting were vascular complications and emergency surgery. All adverse events were reported directly to the London School of Hygiene & Tropical Medicine Clinical Trials Unit and assessed by the primary investigator and clinical lead (appendix pp 28–30).

### Statistical analysis

The expected death rate was calculated from the annual cardiac arrest audit data from the London Ambulance Service (87% mortality with return of spontaneous circulation at any timepoint, 73% mortality with return of spontaneous circulation maintained to hospital), registry data, and the ARREST trial pilot.^9^ Absolute risk reductions of almost 30% were noted from observational registries following implementation of post-arrest treatment bundles.^7^ Observational data in London during trial setup also showed a 31% difference in survival between the cardiac and non-cardiac arrest centres (appendix pp 19–21). Therefore, based on a conservative mortality estimate of 60% (higher mortality providing greater power for an absolute risk reduction) with the combined treatment effect of interventions provided in a cardiac arrest centre, a sample size of 860 patients with 430 in each group would provide 80% power to detect a 10% absolute risk reduction (60–50%) with up to 10% losses to follow-up and a 5% significance level. The data and safety monitoring committee reviewed the data at 25%, 50%, and 75% recruitment without adjusting the final sample size; stringent guidelines were used for the stopping criteria.^17^

The primary analysis was an unadjusted comparison of all-cause mortality at 30 days after randomisation and was performed in patients in the intention-to-treat (ITT) population (all randomly assigned patients for whom consent was not refused) for whom mortality status was known.

Secondary outcomes were analysed in only participants for whom we had available data. A risk ratio (RR) and risk difference, together with a 95% CI and p value, were calculated using a general linear model for binomial outcomes with a log link and identity link function, respectively. Neurological status using the mRS as the primary neurological outcome measure was compared between groups with ordered logistic regression at discharge and 3 months. Treatment effects were measured using proportional odds ratios (ORs). The effect of the COVID-19 pandemic was measured by comparing event rates before and after March 11, 2020, (trial pause date) and an analysis comparing the treatment effect (with 95% CI) presented by time periods (before and after) with an interaction test between time period and treatment from the Cox model. A prespecified analysis adjusting for the following variables was also performed: age, sex, initial shockable rhythm, witnessed cardiac arrest, bystander cardiopulmonary resuscitation, time from cardiac arrest until return of spontaneous circulation, and location of cardiac arrest (private [eg, at home] or public). Multiple imputation by chained equations was used to impute missing values in these variables (20 iterations) and, due to convergence issues when estimating the adjusted RR following imputation, an adjusted OR was estimated using logistic regression. Adverse events were assessed in the ITT population. Statistical analyses were performed using STATA (version 18) and R (version 4.3.0). Full details of the statistical analysis and sample size calculation can be found in pages 5–6 of our Statistical Analysis Plan, provided at the end of the appendix.

The trial was prospectively registered with the International Standard Randomised Controlled Trials Registry, 96585404.

### Role of the funding source

The funders had no role in study design, data collection, data analysis, data interpretation, or writing of the report.

## Results

**Figure 1:**
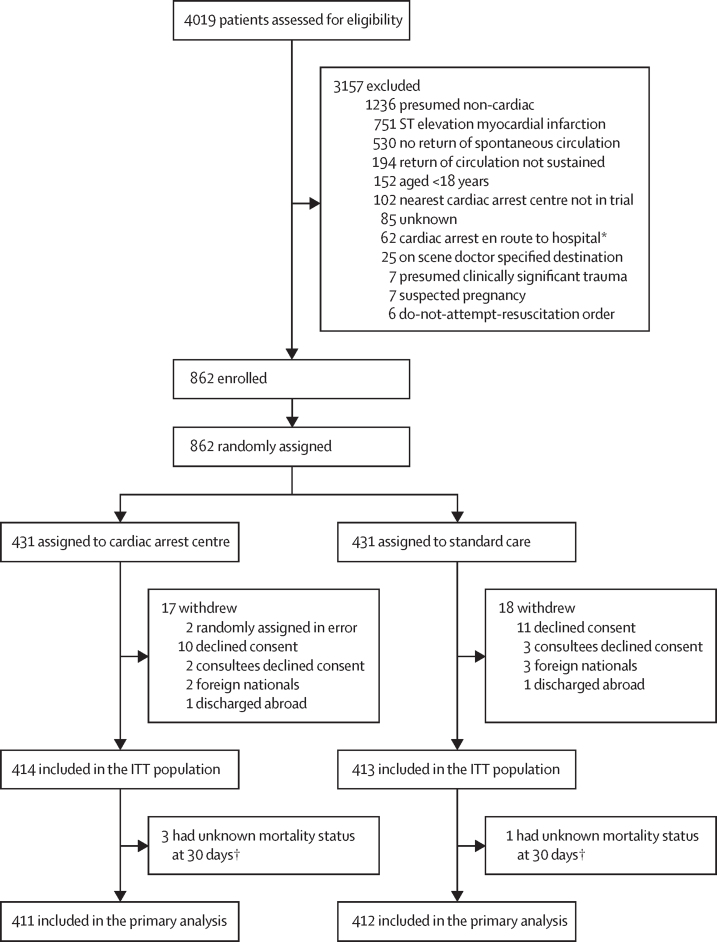
Trial profile ITT=intention-to-treat. *Or cardiac arrrest after the care pathway had been set. †Patients were discharged without follow-up between days 11 and 21.

Between Jan 15, 2018, and Dec 1, 2022, 862 participants were enrolled, of whom 431 (50%) were randomly assigned by London Ambulance Service paramedics to expedited transfer to a cardiac arrest centre and 431 (50%) to standard care (figure 1). 17 participants withdrew from the cardiac arrest centre group and 18 from the standard care group; 414 participants and 413 participants from each group, respectively, were included in the ITT population.

Baseline characteristics of the ITT population, including symptoms before the cardiac arrest, are provided in table 1. Of the 822 participants for whom sex data were obtained, 560 (68%) were male and 262 (32%) were female; data for five participants were unknown. Baseline characteristics were well balanced between the groups. The cause of arrest is provided in the appendix (pp 33–34). A cardiac cause of arrest was identified in 260 (63%) of 414 patients in the cardiac arrest centre group and 245 (59%) of 413 patients in the standard care group. In patients with a cardiac cause of arrest, in the cardiac arrest centre group and the standard care group, coronary disease was identified as the cause in 109 (42%) of 259 patients and 91 (38%) of 242 patients, and an acute coronary cause was identified in 52 (20%) and 45 (19%) patients, respectively. Data on these causes of cardiac arrest for one patient in the cardiac arrest centre group and three in the standard care group could not be obtained. Other frequent causes included primary arrhythmia (85 [33%] of 259 participants in the cardiac arrest centre group and 80 [33%] of 242 participants in the standard care group) and primary cardiomyopathy (45 [17%] of 259 and 46 [19%] of 244). The cause of arrest was not identified in 68 (16%) of 414 participants in the cardiac arrest centre group and 89 (22%) of 413 participants in the standard care group. Key prehospital events and characteristics are summarised in table 2. The median time from cardiac arrest to hospital arrival was 84 min (IQR 68–104) in the cardiac arrest centre group (n=332) and 77 min (63–96) in the standard care group (n=328); a difference of 7 min (95% CI 2–12). All other prehospital characteristics were well balanced between the groups. Hospital treatment and angiographic characteristics are provided in the appendix (pp 35–37).

**Table 1:** Baseline characteristics of the intention-to-treat population

		**Cardiac arrest centre group (n=414)**	**Standard care group (n=413)**
Age, years		63·8 (16)	63·2 (16)
Sex			
	Male	285/412 (69%)	275/410 (67%)
	Female	127/412 (31%)	135/410 (33%)
Ethnicity			
	White	224/414 (54%)	224/413 (54%)
	Asian	69/414 (17%)	69/413 (17%)
	Afro-Caribbean	21/414 (5%)	25/413 (6%)
	Other	39/414 (9%)	45/413 (11%)
	Not known	61/414 (15%)	50/413 (12%)
Medical history			
	Diabetes	98/385 (26%)	90/376 (24%)
	Hypertension	182/376 (48%)	190/372 (51%)
Smoking status			
	Never smoked	96/414 (23%)	83/413 (20%)
	Ex-smoker	50/414 (12%)	53/413 (13%)
	Current smoker	41/414 (10%)	55/413 (13%)
	Not known	227/414 (55%)	222/413 (54%)
Hypercholesterolaemia		99/342 (29%)	83/315 (26%)
Peripheral vascular disease		12/360 (3%)	13/348 (4%)
Cerebrovascular disease		26/369 (7%)	39/362 (11%)
Chronic renal failure		33/375 (9%)	31/362 (9%)
Known ischaemic heart disease		83/362 (23%)	63/353 (18%)
Previous myocardial infarction		54/364 (15%)	48/362 (13%)
Previous percutaneous coronary intervention		46/362 (13%)	34/349 (10%)
Family history of heart disease		32/179 (18%)	32/168 (19%)
Preceding symptoms before cardiac arrest		122/267 (46%)	142/260 (55%)
	Chest pain	29/122 (24%)	43/142 (30%)
	Dizziness	11/122 (9%)	29/142 (20%)
	Breathlessness	50/122 (41%)	49/142 (35%)
	Palpitations	2/122 (2%)	8/142 (6%)
	Other symptoms	61/122 (50%)	74/142 (52%)

Data are mean (SD) or n/N (%). Ethnicity and smoking status had “Not known” as a response category in the case report form and so the denominator for these variables is the total number of patients in the intention-to-treat population; other variables did not have this option, and therefore the denominator for all other variables is the number of patients for whom data were available.

**Table 2:** Prehospital key events in the intention-to-treat population

		**Cardiac arrest centre group (n=414)**	**Standard care group (n=413)**
Location of arrest			
	Private	208 (50%)	242 (59%)
	Public	206 (50%)	171 (41%)
Witnessed arrest			
	Bystander	308 (74%)	307 (74%)
	LAS	30 (7%)	25 (6%)
	Not witnessed	76 (18%)	81 (20%)
Presenting cardiac rhythm			
	AED non-shockable, asystole, or PEA	184 (44%)	188 (46%)
	AED shockable, VF, or pulseless VT	229 (55%)	225 (55%)
	Not known	1 (<1%)	0
Initial CPR attempt			
	Bystander	290 (70%)	313 (76%)
	LAS	123 (30%)	100 (24%)
	Not performed	1 (<1%)	0
Time from arrest to LAS CPR start, min		9 (7–12); n=278	10 (7–12); n=275
First defibrillation performed			
	Public access defibrillator	49 (12%)	54 (13%)
	LAS	211 (51%)	198 (48%)
	Not performed	142 (34%)	150 (36%)
	Not known	12 (3%)	11 (3%)
Time from arrest to first defibrillation, min		10 (7–14); n=194	11 (7–14); n=199
Number of shocks delivered		2 (1–4); n=242	2 (1–3); n=226
Adrenaline administered		267 (65%)	260 (63%)
Adrenaline dose, mg		2 (1–4); n=260	2 (1–4); n=254
Amiodarone administered		68 (16%)	58 (14%)
Amiodarone dose, mg		300 (300–300); n=65	300 (300–300); n=52
Mechanical CPR		100/412 (24%)	93/411 (23%)
Time from arrest to ROSC, mins		24 (15–33); n=310	25 (16–34); n=314
Field termination of resuscitation		2 (1%)	3 (1%)
Time from arrest to hospital arrival, min		84 (68–104); n=332	77 (63–96); n=328
Post-ROSC electrocardiogram*			
	ST-segment elevation	7 (2%)	5 (1%)
	Bundle branch block	116 (28%)	99 (24%)
	ST-segment depression or T wave changes (or both)	156 (38%)	181 (44%)
	No acute changes	91 (22%)	83 (20%)
	No electrocardiogram	44 (11%)	45 (11%)

Data are n (%), median (IQR), or n/N (%). The number of participants for whom data were obtained is presented after median values and n values when the number was less than the total intention-to-teat population. AED=automated external defibrillator. CPR=cardiopulmonary resuscitation. LAS=London Ambulance Service. PEA=pulseless electrical activity. ROSC=return of spontaneous circulation. VF=ventricular fibrillation. VT=ventricular tachycardia.

* The electrocardiogram was reviewed independently after trial enrolment and randomisation. Defibrillation data were analysed for patients with shockable rhythm only.

A higher proportion of patients were identified as being in cardiogenic shock in the cardiac arrest centre group than in the standard care group (112 [28%] of 408 *vs* 93 [23%] of 406). A higher proportion of patients in the cardiac arrest centre group than in the standard care group were admitted to intensive care (330 [80%] of 414 *vs* 286 [69%] of 413), received haemodynamic support (297 [72%] of 412 *vs* 252 [62%] of 406), ventilatory support (353 [86%] of 412 *vs* 312 [76%] of 410), and renal support (46 [11%] of 411 *vs* 34 [8%] of 403). A higher proportion of patients in the cardiac arrest centre group than in the standard care group had coronary angiography (231 [56%] of 412 *vs* 153 [37%] of 410). The median time to coronary angiography was shorter in the cardiac arrest centre group than in the standard care group (2·3 h [IQR 1·7–3·0] *vs* 5·7 h [IQR 3·6–56·0]). The rates of mechanical support device use were well balanced between groups as were the remainder of inpatient characteristics.

**Figure 2:**
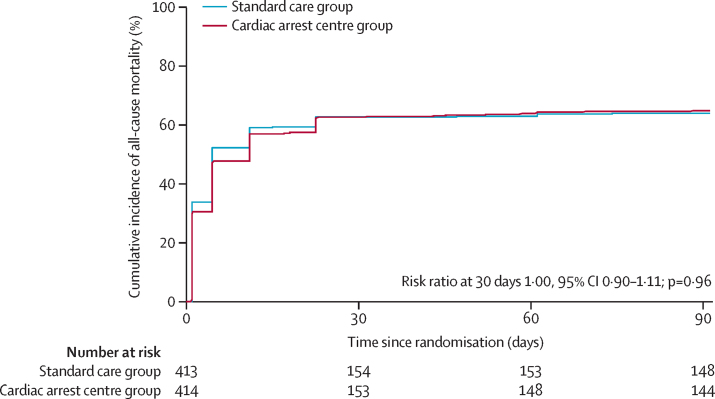
Kaplan-Meier plot of all-cause mortality up to 3 months (intention-to-treat population)

Three participants in the cardiac arrest centre group and one in the standard care group did not have known mortality status at 30 days because they were discharged between days 11 and 21 without follow-up; therefore, primary outcome data were available in 411 (95%) of 431 participants in the cardiac arrest centre group and 412 (96%) of 431 participants in the standard care group (table 3). 30-day all-cause mortality was 258 (63%) of 411 in the cardiac arrest centre group and 258 (63%) of 412 in the standard care group (unadjusted RR for survival 1·00 [95% CI 0·90 to 1·11], p=0·96; risk difference 0·2% [95% CI –6·5 to 6·8]). There was no difference in 3-month all-cause mortality between the two groups (RR 1·02 [95% CI 0·92 to 1·12]; risk difference 1·0% [95% CI –5·6 to 7·5]). The Kaplan-Meier plot for all-cause mortality at 3 months is provided in figure 2.

**Table 3:** Primary and secondary outcomes

		**Cardiac arrest centre group (n=414)**	**Standard care group (n=413)**	**RR, OR, or mean difference (95% CI)**	Adjusted OR*(95% CI) or p value	**Risk difference (95% CI)**
**Primary endpoint**						
30-day mortality		258/411 (63%)	258/412 (63%)	RR 1·00 (0·90 to 1·11)	1·09 (0·73 to 1·63)	0·2% (−6·5 to 6·8)
**Secondary endpoints**						
3-month mortality		267/411 (65%)	263/411 (64%)	RR 1·02 (0·92 to 1·12)	..	1·0% (−5·6 to 7·5%)
mRS score at discharge				OR 1·00 (0·76 to 1·32)	0·99	..
	0	70/413 (17%)	78/402 (19%)	..	..	..
	1	23/413 (6%)	31/402 (8%)	..	..	..
	2	22/413 (5%)	12/402 (3%)	..	..	..
	3	15/413 (4%)	9/402 (2%)	..	..	..
	4	10/413 (2%)	2/402 (1%)	..	..	..
	5	16/413 (4%)	12/402 (3%)	..	..	..
	6	257/413 (62%)	258/402 (64%)	..	..	..
mRS score at 3 months				OR 0·98 (0·73 to 1·31)	0·87	..
	0	75/399 (19)	69/390 (18%)	..	..	..
	1	22/399 (6%)	32/390 (8%)	..	..	..
	2	17/399 (4%)	9/390 (2%)	..	..	..
	3	5/399 (1%)	9/390 (2%)	..	..	..
	4	9/399 (2%)	3/390 (1%)	..	..	..
	5	4/399 (1%)	5/390 (1%)	..	..	..
	6	267/399 (67%)	263/390 (67%)	..	..	..
mRS score at discharge						
	Favourable	130/413 (32%)	130/402 (32%)	RR 1·01 (0·92 to 1·11)	0·79	0·9% (−5·5 to 7·3)
	Unfavourable	283/413 (69%)	272/402 (68%)	..	..	..
mRS score at 3 months						
	Favourable	119/399 (30%)	119/390 (31%)	RR 1·01 (0·92 to 1·11)	0·83	0·7% (−5·7 to 7·1)
	Unfavourable	280/399 (70%)	271/390 (70%)	..	..	..
Mean EQ-5D-5L score		0·68 (0·32); n=97†	0·72 (0·25); n=92†	Mean difference −0·04 (−0·12 to 0·05)	..	..

Data are n/N (%) and mean (SD), unless otherwise specified. Mortality refers to all-cause mortality. mRS=modified Rankin Scale. OR=odds ratio. RR=risk ratio.

* Adjusted OR calculated due to convergence issues.

† The number of participants for whom data were obtained.

**Figure 3:**
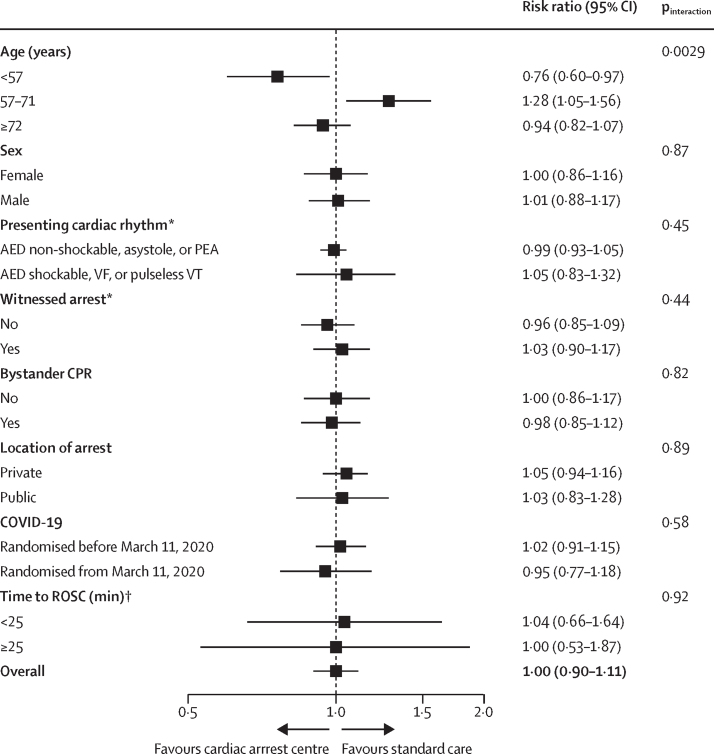
Subgroup analyses for all-cause mortality at 30 days Absolute number of events and total number of participants for each trial arm and subgroup are presented in the appendix (p 45). AED=automated external defibrillator. CPR=cardiopulmonary resuscitation. PEA=pulseless electrical activity. ROSC=return of spontaneous circulation. VF=ventricular fibrillation. VT=ventricular tachycardia. *Due to convergence issues, p_interaction_ values were estimated using Mantel-Haenszel tests. †Following multiple imputation of missing values; due to convergence issues, odds ratios and p_interaction_ values were estimated using logistic regression.

Neurological outcomes were similar at hospital discharge for mRS score (OR 1·00, 95% CI 0·76–1·32) and cerebral performance category score (0·98, 0·74–1·30), and for scores at 3 months (mRS score OR 0·98 [0·73–1·31] and cerebral performance category score OR 0·98 [0·73–1·31); table 3; appendix pp 40–41). Participants who were alive at 3 months had worse neurological outcomes measured with mRS at discharge in the cardiac arrest centre group than in the standard care group (OR 1·55, 1·00–2·41; appendix pp 40–41). There were no between-group differences in EQ-5D-5L score (table 3; appendix p 42). The adjusted analysis for the primary endpoint was consistent with the results of the main analysis (table 3). Subgroup analysis showed that being younger than 57 years was associated with a reduced risk of all-cause mortality by 30 days in the cardiac arrest group (RR 0·76, 95% CI 0·60–0·97; p_interaction_=0·0029**;** figure 3; appendix p 45). However, standard care appeared to favour participants aged 57–71 years in terms of all-cause mortality at 30 days (figure 3). There was no evidence that COVID-19 affected treatment and there were no other important subgroup interactions.

Eight (2%) of 414 patients in the cardiac arrest centre group and three (1%) of 413 in the standard care group has serious adverse events, none of which were deemed related to the trial intervention (appendix p 48).

## Discussion

In this multicentre, randomised trial, we show no difference in the primary endpoint of all-cause mortality at 30 days in patients with resuscitated cardiac arrest without ST elevation in the community who were delivered to a cardiac arrest centre compared with those who were delivered to the geographically closest emergency department. There was no overall difference in neurological outcomes at discharge and at 3 months. Patients in the cardiac arrest centre group had a longer transit time to hospital, were more likely to undergo coronary angiography, and were more likely be admitted to intensive care. Patients in the cardiac arrest centre group had higher rates of cardiogenic shock and an increased requirement for multi-organ support (ventilatory, haemodynamic, and renal).

These findings differ from those in a meta-analysis that found that treatment in a cardiac arrest centre was associated with improved survival with favourable neurological outcomes.^8^ Unlike the present study, these data were almost exclusively observational and, therefore, need to be interpreted with caution due to the risk of unmeasured confounders creating bias. However, it is important to note that some subgroups were identified as benefiting from transfer to a cardiac arrest centre, including patients with the following clinical characteristics: shockable rhythm; absence of return of spontaneous circulation before hospital admission; and return of spontaneous circulation and criteria for ST-elevation myocardial infarction. Although our subgroup analysis of shockable rhythm showed no evidence of an interaction, we had fewer patients than the number included in the meta-analysis.

ARREST is the first and only randomised trial of delivery to a cardiac arrest centre following resuscitated OHCA in the community. The success of this trial is attributable to the research infrastructure at the London Ambulance Service and the pan-London cardiac network, which facilitated the success of a trial of this scale and complexity. Following cardiac arrest of presumed cardiac cause, it would seem intuitive that patients should be transported to a specialist centre with 24-h access to cardiac facilities, specialist intensive care, and other facilities in a well resourced hospital, and that this strategy would improve survival. This assumption is further supported by observational data, including our pre-trial data from patients selected by ambulance crews to be taken to cardiac arrest centres across London, which show that there was a 31·3% absolute percentage point increase in the proportion of patients who survived between those who attended a cardiac arrest centre and those who did not (appendix p 19). Furthermore, recommendations have been published from multiple professional bodies, in the absence of randomised data, that patients should be delivered to a cardiac arrest centre following OHCA.^6^ However, as mentioned above, there are multiple confounders when examining observational data, including selection bias—ie, younger patients with fewer comorbidities will have increased chances of survival whether delivered to a well resourced centre from the community or as an interhospital transfer than older patients with multiple comorbidities. This increased survival benefit was apparent in our pre-trial observational audit data from patients selectively taken to cardiac arrest centres in London; however, when patients were randomly assigned to attend a cardiac arrest centre in our trial, the survival benefit was no longer present**.**

There was no difference in pre-randomisation baseline characteristics, cardiac arrest characteristics, or distribution of coronary disease between the groups. However, there was greater use of advanced organ support and coronary angiography (probably attributable to accessibility) in the cardiac arrest centres than in the standard care group, which did not translate to a difference in mortality. This finding also has resource implications: if multiple interventions do not improve overall survival, they might be better allocated elsewhere. Management of patients after a cardiac arrest can be challenging due to a combination of ischaemia-reperfusion injury and persistence of the underlying cause, preventing or reducing multi-organ dysfunction, and ensuing spiral of cardiogenic shock. In this study we show that delivery of patients resuscitated after cardiac arrest without ST elevation to a nearby emergency department with rapid resuscitation and admission to the local intensive-care unit (with delayed prognostication, temperature control, and haemodynamic support) is as effective, in terms of survival, with favourable neurological outcomes, as transferring them to a large, well resourced, tertiary hospital intensive-care unit. This finding is important for strategic planning of hospital capacity, particularly in the current climate with long hospital waiting lists and planning of post-COVID-19 recovery, and for other emergency work, including trauma, ST-elevation myocardial infarction, and acute aortic dissection that require high-dependency beds and specialist input within these centres. Although the analyses of our secondary outcomes should be considered exploratory, the delay incurred by a slightly longer transit time to hospital (which will be longer outside of London where services are less concentrated) and transfer to a catheter laboratory in a cardiac unit rather than to intensive care directly (where temperature control and haemodynamic stabilisation are available) via the emergency department might be detrimental in terms of neurological outcomes. These findings are consistent with previous randomised trials of early invasive coronary angiography, in which a period of stabilisation was shown to be preferential compared with early intervention.^18^

More patients were in cardiogenic shock in the cardiac arrest centre group than the standard care group. The threshold for treatment might have differed between centres, which was potentially affected by faster access to invasive therapies or was a result of delayed transfer to intensive care. Treatment was not mandated in either group and left to physician discretion, hence the low overall rates of coronary angiography, which most likely reflect the heterogeneity of diagnoses in the non-ST-elevation population, with only 61% of patients with a confirmed cardiac cause of arrest despite presumed cardiac cause on scene; and, of the cardiac causes, approximately 50% were due to primary cardiomyopathy or primary arrhythmia rather than an acute coronary occlusion. Non-shockable rhythm was identified in 45% of patients, which might seem high in a population of patients with return of spontaneous circulation and could reflect the exclusion of patients with ST-segment elevation on the ECG done after return of spontaneous circulation.

In this study, the absence of a difference between the two strategies could be attributed to the individual components of in-hospital management provided in a cardiac arrest centre, including hypothermia, early coronary angiography, and mechanical support, which, in isolation, have not previously shown a survival benefit in an unselected population of patients with cardiac arrest.^18^, ^19^, ^20^ In the absence of these invasive therapies, there might be little difference between the inpatient care provided in a cardiac arrest centre and the care provided in an acute hospital within the UK NHS. This study also highlights the quality of care delivered by the London Ambulance Service and the importance of the first three links in the chain of survival (ie, early recognition, cardiopulmonary resuscitation, and defibrillation) and their effect on outcome.^21^ Only 60% of patients with a presumed cardiac cause of arrest before hospital admission received a final diagnosis of cardiac cause. This finding suggests that it might be challenging to identify patients before hospital admission who would benefit from transfer to a cardiac arrest centre. Prehospital investigations tend to be limited to the post-resuscitation ECG and, in the absence of ST elevation, it can be difficult to establish causation, and it might be preferential for the patient to be delivered to the nearest hospital for diagnosis and inter-hospital transfer to a cardiac arrest centre if deemed appropriate on the basis of the results of further investigations rather than a strategy of delivering all patients with cardiac arrest directly to a specialist centre. However, we acknowledge this strategy might not be possible in rural health-care systems or populations without high-quality acute general hospitals, although it could be argued that based on the findings of this study, resources might be better allocated to raising the minimum standard of acute hospitals than transferring all patients to a cardiac arrest centre.

Although the subgroup findings should be interpreted with caution, the results indicated a possible survival benefit from delivery to a cardiac arrest centre in participants younger than 57 years. In contrast, survival was higher in participants aged 57–71 years with standard care. In the standard care group, mortality was unexpectedly lower in participants aged 57–71 years compared with participants younger than 57 years. Although chance is a likely explanation for these findings, an explanation for the survival benefit in participants younger than 57 years could be due to the pathogenesis of arrest in this younger age group, with greater potential for reversibility and increased physiological reserve. This finding could be an area of future research to establish whether a cohort of younger patients would benefit from this strategy.

The main limitation of this trial was that it was done across London with a dense population in a small geographical area. The London Ambulance Service has rapid response times and short transit times, and delivers high-quality prehospital care, which could limit generalisability to systems with greater geographical spread or longer transit times, and systems in non-urban areas. The discordance between pre-hospital assessment of presumed cardiac cause and definitive cause might have affected patient outcomes in this study. Patients with ST-elevation myocardial infarction were excluded from the study as there is strong evidence to suggest benefit from an early invasive strategy. Participants, ambulance staff, and clinicians were not masked in this study, which might have affected in-hospital treatment strategies and the assessment of neurological outcomes.

In conclusion, this large, multicentre, randomised trial of expedited transfer to a cardiac arrest centre did not show a survival benefit compared with standard of care. This study does not support prehospital transportation of all patients to a cardiac arrest centre following resuscitated cardiac arrest without ST elevation within this health-care setting.

## Data sharing

Individual patient data collected from the study (after de-identification and removal of any data that cannot be shared due to our regulatory agreements) will be made available to other researchers on application to the ARREST trial via email (ARREST@lshtm.ac.uk). A data dictionary, the study protocol, and the statistical analysis plan will also be supplied. These data will be supplied subject to approval of a proposed statistical analysis plan by the ARREST publication committee and to a data access agreement. Data can be shared 12 months after the end of the study (last visit of final patient). This anticipated date is Dec 1, 2024, at the earliest.

## Declaration of interests

We declare no competing interests.
