# Supplementary material for: Expedited transfer to a cardiac arrest centre for non-ST-elevation out-of-hospital cardiac arrest (ARREST): a UK prospective, multicentre, parallel, randomised clinical trial
Source: Lancet. 2023 Oct 14;402(10410):1329–37. doi: 10.1016/S0140-6736(23)01351-X (PMC10877072; doi:10.1016/S0140-6736(23)01351-X)
Supplement: Supplementary appendix [file mmc1.pdf]

THE LANCET

Supplementary appendix

This appendix formed part of the original submission and has been peer reviewed. We post it as supplied by the authors.

Supplement to: Patterson T, Perkins GD, Perkins A, et al. Expedited transfer to a cardiac arrest centre for non-ST-elevation out-of-hospital cardiac arrest (ARREST): a UK prospective, multicentre, parallel, randomised clinical trial. *Lancet* 2023; published online Aug 27. https://doi.org/10.1016/S0140-6736(23)01351-X.

Appendix

Table of Contents

ARREST TRIAL COLLABORATORS **3**

LONDON AMBULANCE SERVICE EMERGENCY RESPONSE **8**

RANDOMISATION PROCESS **10**

PARAMEDIC RANDOMISATION 10

RANDOMISATION WEBSITE 10

BACKGROUND AND METHODS **11**

FIGURE 1: ALL ACUTE HOSPITALS IN LONDON PARTICIPATING IN ARREST 11

TABLE 1: EMERGENCY FACILITIES IN CARDIAC ARREST CENTRES AND NON-CARDIAC ARREST CENTRES 13

TABLE 2. MINIMUM CRITERIA FOR CARDIAC ARREST CENTRES 18

TABLE 3: OUT-OF-HOSPITAL CARDIAC ARREST VOLUME AND SURVIVAL IN LONDON PER CENTRE (2017-18). 19

TABLE 4: TRIAL PROCEDURES AND OUTCOME ASSESSMENT 22

INCLUSION AND EXCLUSION CRITERIA 24

CONFIDENTIALITY ADVISORY GROUP 25

RANDOMISATION PROCEDURES 26

Access to randomisation site *26*

SAFETY REPORTING 28

Definition *28*

Expected serious adverse events related to usual clinical care *28*

Unexpected serious adverse events *28*

Unexpected non-serious adverse events *29*

Reporting unexpected adverse events *29*

Assessment of intensity *29*

Assessment of causality ... *30*

Hospital Referral Pathways in London ... 31

RESULTS ... 32

Protocol Deviations and Crossover ... 32

Table 5: Cause of arrest in the trial population ... 33

Table 6: Inpatient and angiographic characteristics ... 35

Table 7: Cerebral Performance Category (CPC) in the ITT population ... 38

Table 8: Neurological outcome restricted to those surviving at 3 months ... 40

Table 9: EQ-5D-5L at discharge ... 42

Table 10: Subgroup analyses for all-cause mortality at 30 days (ITT population) ... 45

Table 11: Adverse events in the ITT population ... 48

ARREST Trial Collaborators

**Ambulance Service**	**Principal Investigator**	**Co-Investigators**
London Ambulance Service	Prof Rachael Fothergill	Adam Mellett-Smith, Anthony Lampard, Desiree Papadopoulos Johanna Hughes, Valentina Pendolino, Joanna Shaw, Clara Bannister, Amy Long, Justin Kearney, Gabriel Palti, Joanne Ritches-Price, Mark Whitbread (former)
Cardiac Arrest Centres	Principal Investigator	Co-Investigators
St Thomas Hospital	Dr Tiffany Patterson	Prof Simon Redwood, Prof Divaka Perera, Dr James Coutts, Dr Brian Clapp, Prof Bernard Prendergast
Barts Heart Centre	Dr Ajay Jain	Dr Andrew Wragg
King’s College Hospital	Professor Philip MacCarthy	Dr Jonathan Byrne
Harefield Hospital	Dr Miles Dalby	
St George’s Hospital	Dr Sami Firoozi	
Royal Free Hospital	Dr Roby Rakhit	

Hammersmith Hospital	Dr Iqbal Malik	
Non-CAC	Principal Investigator	Co-Investigators
Barnet Hospital	Dr Roby Rakhit	
Northwick Park Hospital	Dr Nigel Stephens	
Hillingdon Hospital	Dr Gareth Rosser	
Queens Hospital, Romford	Dr Darryl Wood	
University College Hospital	Dr Robert Bell	
Homerton Hospital	Dr Arvinder Kurbaan	
Ealing Hospital	Dr Nigel Stephens	
Queen Elizabeth Hospital	Dr Antonis Pavlidis	
North Middlesex Hospital	Dr Muhiddin Ozkor	
West Middlesex Hospital	Dr Sukhjinder Nijjer	
Whittington Hospital	Dr Rita Das	
Kingston Hospital	Therese Sidney	
University Hospital Lewisham	Dr Antonis Pavlidis	
St Helier Hospital	Dr Richard Bogle	
Newham Hospital	Dr Ajay Jain	
St Mary’s Hospital	Dr Iqbal Malik	
King George Hospital	Dr Darryl Wood	
Charing Cross Hospital	Dr Iqbal Malik	
Chelsea and Westminster Hospital	Dr Patrick Roberts	

Princess Royal Hospital	Dr Ian Webb	
Croydon University Hospital	Dr Oliver Spencer	
Darent Valley Hospital	Dr Edward Petzer	
Watford Hospital	Dr Masood Khan	
Royal London Hospital	Dr Ajay Jain	
Whipps Cross Hospital	Dr Ajay Jain	
Central Middlesex Hospital	Dr Nigel Stephens	
Chase Farm Hospital	Dr Roby Rakhit	
East Surrey Hospital	Dr Maciej Marciniak	
Trial Steering Committee Membership	Role	
Mark De Belder	Chair	
Lucy Blows	Member	
Nick Curzen	Member	
Dawn Adamson	Member	
Mamas Mamas	Member	
Martin Brown	Member	
Garth Lane	Patient representative	
Michael Connor	Patient representative	
Tiffany Patterson	Non-independent member	
Tim Clayton	Non-independent member	
Simon Redwood	Non-independent member	

Joanne Nevett	Non-independent member
Mark Whitbread (former)	Non-independent member
Data Safety Monitoring Committee Membership	Role
Rod Stables	Chair
Tim Morris	Independent statistician
Keith Muir	Independent member
Douglas Chamberlain (former)	Independent member
Project Management Group	Centre
Simon Redwood	Guy's and St Thomas NHS Foundation Trust
Tiffany Patterson	Guy's and St Thomas NHS Foundation Trust
Karen Wilson	Guy's and St Thomas NHS Foundation Trust
Alexander Perkins	London School of Hygiene and Tropical Medicine CTU
Richard Evans	London School of Hygiene and Tropical Medicine CTU
Tim Clayton	London School of Hygiene and Tropical Medicine CTU
Jerry Nolan	Royal United Hospital Bath
Gavin Perkins	University of Warwick Clinical Trials Unit
Rachael Fothergill	CARU London Ambulance Service
Adam Mellett-Smith	CARU London Ambulance Service
LSHTM Clinical Trials Unit	Role
Tim Clayton	Senior Statistician

Matt Dodd	Statistician
Matthew Kwok	Trial Manager
Alexander (Addi) Perkins	Trial Manager (former)
Steven Robertson	Senior Data Manager
Richard Evans	Senior Manager
Megan Knight	Assistant Trial Manager
Lauren Jerome	Assistant Trial Manager (former)

CTU Clinical Trials Unit, CARU Clinical Audit and Research Unit

London Ambulance Service Emergency Response

Ambulance services in the UK are activated through a central emergency phone number (999), which based on geographical location directs the caller to the nearest emergency medical service responder. London and the Greater London area is served by London Ambulance Service NHS Trust (LAS). The London Ambulance Service is the primary provider of prehospital emergency care across Greater London is covering an area of 620 square miles, serving an estimated 9 million people. London Ambulance Service is one of the busiest ambulance services in the world, with over 3,500 frontline clinicians providing a face-to-face response to more than 1.2 million patients each year, over 12,000 of which are out of hospital cardiac arrest. Emergency calls are triaged by Emergency Call Handlers using the advanced Medical Priority Dispatch System (MPDS; IAED, Salt Lake City, Utah, USA). As well as gathering details about the patient and the incident, Call Handlers also deliver CPR instructions to the caller. Ambulance resources are concurrently dispatched during this time. Out-of-hospital cardiac arrests receive the highest priority response with a minimum of two vehicles (one capable of transporting the patient) being immediately dispatched to the scene, carrying at least one advanced life support trained paramedic. All emergency vehicles are staffed by paramedics or non-paramedic clinicians (e.g. emergency medical technicians) trained in cardiopulmonary resuscitation who are able to interpret 12-lead electrocardiograms. All ambulance crews follow resuscitation guidelines when resuscitating patients, which is in accordance with European Resuscitation Council guidance. Defibrillation is undertaken in AED mode in line with local procedures. Current national guidance for response times for a cardiac arrest require a defibrillator capable vehicle to arrive within 7

minutes (in three quarters of cases) and an ambulance to arrive within 30 minutes. Based on London Ambulance Service cardiac arrest annual audit data, resuscitation is attempted in approximately 36% of all out-of-hospital cardiac arrests. Resuscitation can be withheld if there is unequivocal evidence of death or in cases where a do not attempt resuscitation order or other valid advanced directive is present. If attempts at resuscitation are unsuccessful despite advanced life support interventions, resuscitation may be withdrawn on scene in line with national guidelines. Out-of-hospital cardiac arrest patients are only routinely conveyed to hospital if return of spontaneous circulation is achieved or where there is a clear reversible cause requiring hospital intervention.

The Advanced Paramedic Practitioner (APP) model is specific to LAS and APPs receive additional education and critical care training and are targeted to the most critically ill and injured patients. Working as solo clinicians in a fast response car, APPs provide on-scene leadership, undertake advanced decision making and provide a range of enhanced interventions with a wider drug formulary. A maximum of 5 APPs are operational across Greater London at any one time. The APP operating model also includes the placement of an additional APP within with the Emergency Call Centre to identify those suitable for a targeted APP response, which includes OHCAs were resuscitation is appropriate.

Randomisation Process

Paramedic randomisation

Randomisation was performed by designated LAS staff at the Advanced Paramedic Practitioner (APP) dispatch desk. Paramedics attending a suspected OHCA assessed the patient for eligibility, once confirmed, the on-site paramedic asked the APP desk to randomise the patient into the trial. The on-site paramedics provided the necessary patient details required to complete randomisation. The APP desk staff then accessed the randomisation site, entered the required details, and generated a study ID and treatment allocation. Patients were randomised either to an expedited transfer to a cardiac arrest centre or to receive standard of care. The APP desk then informed the on-site paramedic which group the patient had been randomised to, and the on-site paramedic proceeded as appropriate. Following this, the APP desk provided the necessary information to the Clinical Audit and Research Unit at London Ambulance Service to enable them to track the patient's clinical record to gain clinical data.

Randomisation Website

Access to the randomisation site www.sealedenvelope.com was strictly controlled and available only to delegated staff of the APP desk at London Ambulance Service that had received appropriate training. Each staff member had a unique account for accessing the randomisation site, and did not share these details of their account with other staff members. APP desk staff logged into their accounts at the beginning of each shift and remain logged in for the duration of the shift. Each session timed out after 12 hours.

Background and Methods

Figure 1: All acute hospitals in London participating in ARREST

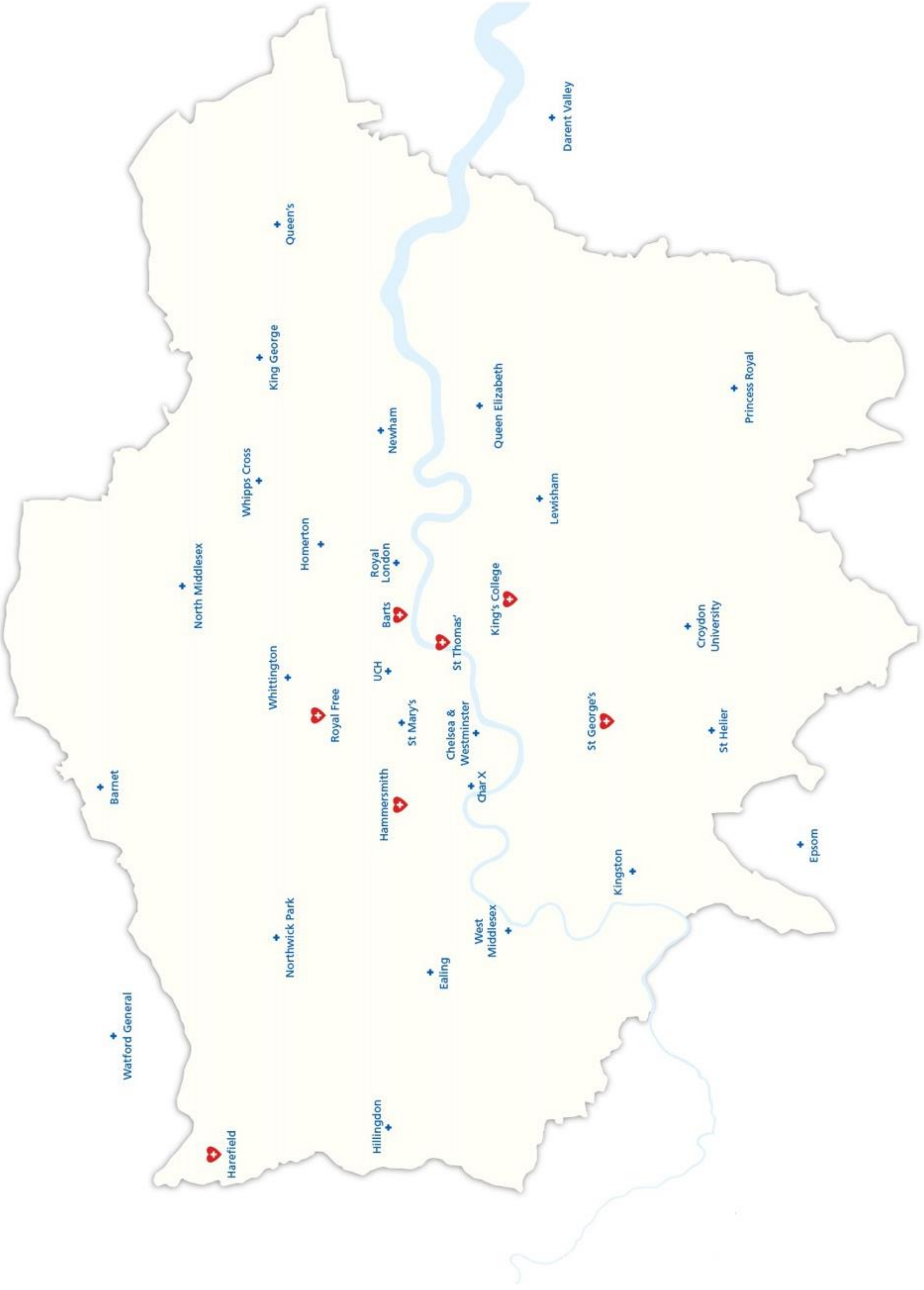

This map of London identifies all acute hospitals receiving patients from London Ambulance Service and were therefore ARREST trial receiving hospitals (sites). Seven of these hospitals were designated as cardiac arrest centres (red hearts) as they already provided 24/7 access to interventional cardiology, cardiac surgery and cardiac intensive care facilities. These centres are referral centres for the non-specialist centres for specialist cardiac care including cardiogenic shock (right and left heart failure), percutaneous interventions for structural heart disease, percutaneous complex coronary intervention, cardiac surgery including emergency aortic dissection surgery or non-cardiac surgery requiring specialist cardiac support. Four of the cardiac arrest centres also had emergency departments, three did not. Twenty-eight hospitals had emergency departments without access to 24/7 onsite interventional cardiology and specialist intensive care facilities and would refer or transfer patients to their specialist cardiac arrest centre as required.

Table 1: Emergency facilities in cardiac arrest centres and non-cardiac arrest centres

Cardiac Arrest Centre	Echo	Pacing	Coronary angiography+/-PCI	Haemodynamic support	Cardiac surgery	Vascular surgery	Neurology and neurosurgery
St Thomas Hospital	Yes	Yes	Yes	VA ECMO, IABP, Impella	Yes	Yes	Access via King's College Hospital
Barts Heart Centre	Yes	Yes	Yes	VA ECMO, IABP, Impella	Yes	No	Access via Royal London Hospital
King's College Hospital	Yes	Yes	Yes	VA ECMO, IABP, Impella	Yes	Yes	Yes
Harefield Hospital	Yes	Yes	Yes	VA ECMO, IABP, Impella	Yes	No	No

St George's Hospital	Yes	Yes	Yes	VA ECMO, IABP, Impella	Yes	Yes	Yes
Royal Free Hospital	Yes	Yes	Yes	IABP	Yes	Yes	Access via University College Hospital
Hammersmith Hospital	Yes	Yes	Yes	IABP, Impella	Yes	No	Access via Charing Cross hospital
Non-CAC							
Barnet Hospital	No	No	No	No	No	No	No
Northwick Park Hospital	No	No	No	No	No	No	No
Hillingdon Hospital	No	No	No	No	No	No	No
Queens Hospital, Romford	No	No	No	No	No	No	No
University College Hospital	No	No	No	No	No	No	Yes

Homerton Hospital	No	No	No	No	No	No	No
Ealing Hospital	No	No	No	No	No	No	No
Queen Elizabeth Hospital	No	No	No	No	No	No	No
North Middlesex Hospital	No	No	No	No	No	No	No
West Middlesex Hospital	No	No	No	No	No	No	No
Whittington Hospital	No	No	No	No	No	No	No
Kingston Hospital	No	No	No	No	No	No	No
University Hospital Lewisham	No	No	No	No	No	No	No
St Helier Hospital	No	No	No	No	No	No	No
Newham Hospital	No	No	No	No	No	No	No
St Mary's Hospital	No	No	No	No	No	Yes	No
King George Hospital	No	No	No	No	No	No	No
Charing Cross Hospital	No	No	No	No	No	No	Yes

Chelsea & Westminster Hospital	No	No	No	No	No	No	No
Princess Royal Hospital	No	No	No	No	No	No	No
Croydon University Hospital	No	No	No	No	No	No	No
Darent Valley Hospital	No	No	No	No	No	No	No
Watford Hospital	No	No	No	No	No	No	No
Royal London Hospital	No	No	No	No	No	No	Yes
Whipps Cross Hospital	No	No	No	No	No	No	No
Central Middlesex Hospital	No	No	No	No	No	No	No
Chase Farm Hospital	No	No	No	No	No	No	No
East Surrey Hospital	No	No	No	No	No	No	No

This table provides information regarding emergency out-of-hours on-site facilities per centre at the time of trial setup. In-hours (9am to 5pm) facilities varied considerably between hospitals, with variable access to echocardiography, diagnostic angiography only or angiography and percutaneous coronary intervention (PCI). Most commonly, patients requiring coronary angiography would be transferred from a non-cardiac arrest centre to one of the 7 cardiac arrest centres that were PCI-capable hospitals.

Table 2. Minimum criteria for Cardiac Arrest Centres

Specialty	Services and interventions
General	Standardised post-resuscitation protocol
Critical Care	Airway management and ventilatory support
	Hemodynamic and renal support
	Acid-base, electrolyte and glucose control
	Targeted temperature management
Cardiothoracic	Echocardiography
	Immediate coronary angiography and revascularisation strategies*
	Mechanical circulatory support
	Electrophysiology and heart failure specialist input ± device insertion
Radiology	24 hour imaging and interpretation including computed tomography to rule in/out respiratory or neurological causes
Neurology	Seizure control
	Standardized multimodality neuroprognostication (including neurophysiology testing)
	Physiotherapy and referral to neurorehabilitation services

Table 3: Out-of-hospital cardiac arrest volume and survival in London per centre (2017-18).

Cardiac Arrest Centre	Number of patients	Survival
	Median 125 (IQR 100 to 167)	Mean 45.7% (±0.1)
St Thomas Hospital	112	47.8% (32/67)
Barts Heart Centre	125	55.8% (53/95)
King's College Hospital	189	36.5% (46/126)
Harefield Hospital	61	54.2% (26/48)
St George's Hospital	184	36.4% (47/129)
Royal Free Hospital	150	42.5% (45/106)
Hammersmith Hospital	88	47% (31/66)
Non-CAC	Number of patients	Survival
	Median 62 (IQR 44 to 79)	Mean 14.4% (±0.1)
Barnet Hospital	50	26.1% (6/23)
Northwick Park Hospital	110	9.6% (5/52)
Hillingdon Hospital	68	15.8% (6/38)
Queens Hospital, Romford	119	9.6% (5/52)
University College Hospital	34	26.7% (4/15)
Homerton Hospital	44	4.8% (1/21)
Ealing Hospital	56	16.7% (5/30)

Queen Elizabeth Hospital	107	15.9% (7/44)
North Middlesex Hospital	107	17.3% (9/52)
West Middlesex Hospital	78	9.4% (3/32)
Whittington Hospital	32	18.8% (3/16)
Kingston Hospital	64	16.1% (5/31)
University Hospital Lewisham	58	17.2% (5/29)
St Helier Hospital	44	10% (2/20)
Newham Hospital	80	10.3% (3/29)
St Mary's Hospital	70	25.7% (9/35)
King George Hospital	57	4.8% (1/21)
Charing Cross Hospital	34	9.1% (1/11)
Chelsea & Westminster Hospital	33	27.8% (5/18)
Princess Royal Hospital	59	3.1% (1/32)
Croydon University Hospital	69	14.7% (5/34)
Darent Valley Hospital	11	0% (0/4)
Watford Hospital	Unavailable	Unavailable
Royal London Hospital	86	18.4% (7/38)
Whipps Cross Hospital	76	16.7% (5/30)
Central Middlesex Hospital	Unavailable	Unavailable
Chase Farm Hospital	Unavailable	Unavailable
East Surrey Hospital	Unavailable	Unavailable

This table demonstrates the destination of resuscitated out-of-hospital cardiac arrests across London (2017-2018). Cardiac arrest centres received on average twice as many cardiac arrests with a median of 125 patients (interquartile range 100 to 167) compared to non-cardiac arrest centres which received a median of 62 patients (interquartile range 44 to 79). These data represent patients with return of spontaneous circulation maintained to hospital. Survival data is provided on patients with known outcomes as the denominator. There is 31% difference in survival between cardiac arrest centres and non-cardiac arrest centres based on these observational data. This report encompasses all patients following cardiac arrest, not just those with presumed cardiac cause and includes both ST-elevation and non-ST-elevation patients. Data obtained from London Ambulance Service Cardiac Arrest Report 2017-2018.

Table 4: Trial procedures and outcome assessment

	Pre-hospital	Hospital arrival	In-hospital	30 day	3 month	6 month	12 month
Review of eligibility criteria	X						
ROSC assessment	X	X					
Randomisation	X						
Transfer to cardiac arrest centre or control	X						
PIS & Informed consent, Personal or Professional Consultee			X				
Neurological status			X		X		
Mortality status		X	X	X	X	X	X
EQ-5D-5L				X			
Service use questionnaire				X	X		
SAE / NSAEs	X	X	X	X	X		

Pre-hospital assessments and trial procedures were performed by LAS ambulance staff trained in trial procedures. Collection of baseline characteristics and cardiac arrest characteristics was performed by LAS research paramedics and obtained from the ambulance patient report form (clinical log) until the patient was consented. If the patient died before consent was obtained, mortality data was collected by research paramedics. Delegated cardiovascular research nurses collected in-hospital care data and the 30-day and 3-month follow up time points. Delegated research nurses at St Thomas' collected mortality data for the 6-month and 12-month follow-up time points through central mortality tracking via the Office of National Statistics. Abbreviations: ROSC return of spontaneous circulation, PIS patient information sheet, SAE serious adverse event or non-serious adverse event assessment

Inclusion and Exclusion Criteria

Inclusion criteria

Patients must meet *all* of the following criteria:

Out-of-hospital cardiac arrest (OHCA)

Return of spontaneous circulation (ROSC)

Age 18 or over (known or presumed)

Exclusion criteria

Patients will be excluded if they meet *any* of the following criteria:

Criteria for ST-elevation myocardial infarction on 12-Lead electrocardiogram (ECG)

Do Not Attempt Resuscitation (DNAR) Order

Cardiac arrest suffered after care pathway set and patient en route

Suspected pregnancy

Presumed non-cardiac cause (for example; trauma, drowning, suicide, drug overdose)

Presumed significant trauma/injury

Confidentiality advisory group

Due to the nature of the trial, there are three possible instances where patient identifiers in the absence of consent will need to be accessed by researchers.

1. During the identification process LAS research paramedics will access LAS records in order to identify patients.
2. Patients who are entered into the trial but do not regain the capacity to consent and die shortly after enrolment before a consultee declaration can be gathered. In these cases it is key that the trial is able to retain the non-identifiable data for analysis.
3. Patients for whom there is no consent or consultee declaration, have been transferred to another hospital and have not replied to multiple contact attempts from the research team. In these cases it is key that the trial is able to track mortality data on these patients for the primary endpoint and retain non-identifiable data for analysis.

Permission has been granted by CAG to allow the use of identifiable data as outlined above.

The CAG reference number is 17/CAG/0151

Randomisation procedures

Designated LAS staff at the Advanced Paramedic Practitioner (APP) dispatch desk will randomise patients into the intervention arm or control arm using the following procedure:

- Paramedics attending a suspected OHCA will assess the patient for eligibility. Once eligibility is confirmed, the on-site paramedic will ask the APP desk to randomise the patient into the trial.
- The on-site paramedics will call the APP dispatch desk to provide the patient details required to complete randomisation.
- The APP desk staff will access the randomisation site, enter the required details, and generate a study ID and treatment allocation. Patients will be randomised either to an expedited transfer to a CAC (see section 10.1) or to receive standard of care (see section 10.2).
- The APP desk will inform the on-site paramedic which group the patient has been randomised to, and the on-site paramedic will proceed as appropriate.
- The APP desk will provide the necessary information to the Clinical Audit and Research Unit (CARU) at LAS for them to track the patient report form (PRF) to gain clinical data.

Access to randomisation site

Access to the randomisation site sealedenvelope.com will be strictly controlled and available only to delegated staff of the APP desk at LAS that have received appropriate training. Delegation and training logs will be recorded both at LAS and at the LSHTM CTU. Each staff member will have a unique account for accessing the randomisation site, and will not share these details of their account with other staff members.

If a staff member is unable to access their account, they should contact the LSHTM CTU to request an account reset. APP desk staff will log in to their accounts at the beginning of each shift and remain logged in for the duration of the shift. Each session will time out after 12

hours. The daily login to the account will mitigate the risk of staff forgetting their login details and losing access to the randomisation service.

Safety reporting

Definition

Events that are collected on the electronic case report form (eCRF) or are part of the usual complications post cardiac arrest do not need to be reported for this trial. Unexpected adverse events should however be reported to the ARREST CTU.

Safety reporting for each patient should commence from time of randomisation to completion of follow up at one year after randomisation.

Expected serious adverse events related to usual clinical care

These events are recognised complications of cardiac arrest. They will be recorded on the eCRF but do not need to be reported separately on an SAE form:

1. Death
2. Myocardial Infarction
3. Stroke
4. Neurological complications
5. Multi-organ failure

The following are considered expected adverse events for cardiac arrest patients undergoing routine clinical care and as such do not need to be reported:

1. Vascular complications
2. Emergency surgery

Unexpected serious adverse events

Any untoward medical occurrence/effect that:

1. Results in death
2. Is life-threatening*
3. Requires hospitalisation or prolongation of existing inpatient's hospitalisation

4. Results in persistent or significant disability or incapacity

**Life-threatening* in the definition of a serious adverse event refers to an event in which the patient was at risk of death at the time of event; it does not refer to an event which hypothetically might have caused death if it were more severe.

SAEs should be reported to the CTU within 7 days. The report should include an assessment of causality by the Principal Investigator (PI) at each site. The Chief Investigator (CI) will be responsible for the prompt notification of findings that could adversely affect the health of patients or impact on the conduct of the trial. Notification of confirmed unexpected and related SAEs will be to the sponsor, the Research Ethics Committee (REC) and the Data and Safety Monitoring Committee (DSMC).

Unexpected non-serious adverse events

The PI or research nurse should evaluate unexpected non-serious adverse events. This should include an assessment of causality and intensity and reports made within 14 days. The CTU will keep detailed records of all unexpected adverse events reported. The CI will review reports to consider intensity, causality and expectedness. As appropriate, these will be reported to the sponsor, the DSMC and the REC.

Reporting unexpected adverse events

Investigators will make their reports of all unexpected adverse events, whether serious or not, to the ARREST CTU by email to arrest@LSHTM.ac.uk or by secure fax to 020 7927 2189.

Assessment of intensity

Mild: The patient is aware of the event or symptom, but the event or symptom is easily tolerated.

Moderate: The patient experiences sufficient discomfort to interfere with or reduce his or her usual level of activity.

Severe: Significant impairment of functioning; the patient is unable to carry out usual activities and/or the patient's life is at risk from the event.

Assessment of causality

Probable: A causal relationship is clinically / biologically highly plausible and there is a plausible time sequence between onset of the adverse event and the treatment

Possible: A causal relationship is clinically / biologically plausible and there is a plausible time sequence between onset of the adverse event and the treatment

Unlikely: A causal relationship is improbable and another documented cause of the adverse event is most plausible.

Unrelated: A causal relationship can definitely be excluded and another documented cause of the adverse event is most plausible.

Hospital Referral Pathways in London

The designated cardiac arrest centres in London are also receiving hospitals for the acute general hospitals in their catchment area. Every acute general hospital has a designated cardiac centre (one of the 7 cardiac arrest centres in Figure 1) providing 24/7 on-site tertiary level cardiology and intensive care in addition to on-site cardiac surgery support to these general hospitals as part of the larger National Health Service network. Referrals can be made during normal working hours or out of hours with various degrees of urgency depending on the acuity of the presentation. These are on the whole physician to physician referrals (on call bleep/phone service overnight) for specialist care not within the remit of the local hospital and transfers can range from technician assisted transportation, to anaesthetic supported transport and even ECMO retrieval systems for patients in refractory cardiogenic shock.

Results

Protocol Deviations and Crossover

19 protocol deviations were recorded:

- 3 cross-overs
  - 1 participant randomised to ED, taken to CAC
  - 2 participants randomised to CAC, taken to ED
- 1 patient transferred to a site which had not yet opened to recruitment
- 10 patients did not meet the eligibility criteria, for the following reasons;
  - 4 randomised when patient was considered probable non-cardiac cause
  - 1 randomised while attending outpatient clinic at hospital (considered in-hospital cardiac arrest)
  - 5 Misinterpretation of ECG, STEMI confirmed
  - 2 participants not taken to the geographically closest ED
  - 2 randomised in error

Table 5: Cause of arrest in the trial population

	Cardiac arrest centre	Standard care
	(n=414)	(n=413)
Cause of arrest*		
Cardiac	260/414 (62.8)	245/413 (59.3)
Non-cardiac	86/414 (20.8)	79/413 (19.1)
Not known	68/414 (16.4)	89/413 (21.5)
Primary arrhythmia	85/259 (32.8)	80/242 (33.1)
Brugada syndrome	0/85 (0.0)	1/80 (1.3)
Idiopathic VF	46/85 (54.1)	33/80 (41.3)
Long QT syndrome	2/85 (2.4)	1/80 (1.3)
Other	37/85 (43.5)	45/80 (56.3)
Primary cardiomyopathy	45/259 (17.4)	46/244 (18.9)
Arrhythmogenic cardiomyopathy	3/45 (6.7)	7/46 (15.2)
Dilated cardiomyopathy	22/45 (48.9)	17/46 (37.0)
Hypertrophic cardiomyopathy	7/45 (15.6)	12/46 (26.1)
Other	13/45 (28.9)	10/46 (21.7)
Coronary artery disease	109/259 (42.1)	91/242 (37.6)
With acute occlusion/ruptured plaque	52/107 (48.6)	45/81 (55.6)
Without acute occlusion but >50% obstruction in one or more vessels	43/107 (40.2)	25/81 (30.9)

Without acute occlusion but known prior ischemic scar	12/107 (11.2)	11/81 (13.6)
Coronary spasm	2/259 (0.8)	2/242 (0.8)
Tamponade	0/259 (0.0)	2/242 (0.8)
Vascular (including dissection)	4/259 (1.5)	6/242 (2.5)
Thoracic aorta	1/4 (25.0)	1/6 (16.7)
Other	3/4 (75.0)	5/6 (83.3)
Valvular heart disease	9/259 (3.5)	13/242 (5.4)
Inflammatory myocarditis/pericarditis	4/259 (1.5)	5/242 (2.1)
Infective	2/4 (50.0)	0/5 (0.0)
Non-specific	1/4 (25.0)	3/5 (60.0)
Other	1/4 (25.0)	2/5 (40.0)
Other cause	64/258 (24.8)	66/240 (27.5)

*Determined by receiving hospital

Abbreviations: VF ventricular fibrillation, data presented as n/N (%)

Table 6: Inpatient and angiographic characteristics

	Cardiac arrest centre n=414	Standard care n=413
Coronary angiogram performed	231/412 (56.1)	153/410 (37.3)
Arrest to coronary angiography (hours), median (IQR)	2.3 (1.7 to 3.0), n=198	5.7 (3.6 to 56.0), n=134
Inpatient transfer to cardiac arrest centre*	-	70/406 (17.2)
Severity of coronary artery disease		
No significant disease	91/203 (44.8)	55/125 (44.0)
1-vessel	34/203 (16.7)	23/125 (18.4)
2-vessel	31/203 (15.3)	26/125 (20.8)
3-vessel	47/203 (23.2)	21/125 (16.8)
Revascularisation performed	83/231 (35.9)	65/153 (42.5)
Type of revascularisation		
CABG	11/83 (13.3)	7/65 (10.8)
PCI	72/83 (86.7)	58/65 (89.2)
If PCI, vessel treated		
Left main stem	5/72 (6.9)	4/57 (7.0)
Left anterior descending	39/72 (54.2)	27/57 (47.4)
Circumflex	13/72 (18.1)	13/57 (22.8)
Right coronary	15/72 (20.8)	12/57 (21.1)
Graft	0/72 (0.0)	1/57 (1.8)

Number of vessels treated		
None	0/72 (0.0)	1/58 (1.7)
1-vessel	56/72 (77.8)	40/58 (69.0)
2-vessel	11/72 (15.3)	13/58 (22.4)
3 or more-vessel	5/72 (6.9)	4/58 (6.9)
Cardiogenic shock	112/408 (27.5)	93/406 (22.9)
Mechanical circulatory support device		
Intra-aortic balloon pump	27/412 (6.6)	21/403 (5.2)
Impella device	1/412 (0.2)	3/405 (0.7)
ECMO	3/412 (0.7)	2/404 (0.5)
Not inserted	383/412 (93.0)	376/402 (93.5)
Organ support		
Ventilatory	353/412 (85.7)	312/410 (76.1)
Renal	46/411 (11.2)	34/403 (8.4)
Hemodynamic (inotropes)	297/412 (72.1)	252/406 (62.1)
Complications		
Reinfarction	9/411 (2.2)	6/408 (1.5)
Target vessel revascularisation	7/412 (1.7)	4/408 (1.0)
Sepsis	31/411 (7.5)	31/408 (7.6)
Stroke	15/412 (3.6)	15/410 (3.7)
Moderate or severe bleeding (BARC 3 or more)	12/408 (2.9)	9/406 (2.2)

*Permitted in trial protocol. Abbreviations: IQR interquartile range, CABG coronary artery bypass graft surgery, PCI percutaneous coronary intervention, ECMO extracorporeal

membrane oxygenation, BARC bleeding academic research consortium criteria. Data presented as n/N (%) or median and IQR

Table 7: Cerebral Performance Category (CPC) in the ITT population

	Cardiac arrest centre	Standard care	Effect estimate (95% CI)	p-value
CPC at discharge (observed data)				
1	102/411 (24.8)	109/401 (27.2)		
2	24/411 (5.8)	19/401 (4.7)		
3	18/411 (4.4)	9/401 (2.2)		
4	10/411 (2.4)	6/401 (1.5)		
5	257/411 (62.5)	258/401 (64.3)	0.98 (0.74 to 1.30)	0.912
CPC at 3 months (observed data)				
1	105/400 (26.3)	102/390 (26.2)		
2	14/400 (3.5)	15/390 (3.8)		
3	11/400 (2.8)	7/390 (1.8)		
4	3/400 (0.8)	3/390 (0.8)		
5	267/400 (66.8)	263/390 (67.4)	0.98 (0.73 to 1.31)	0.883
CPC at discharge (observed data)				
Favourable	126/411 (30.7)	128/401 (31.9)	1.02 (0.93 to 1.12)	0.698

Unfavourable	285/411 (69.3)	273/401 (68.1)	1.3 (-5.1 to 7.6)	0.698
CPC at 3 months<br><br>(observed data)				
Favourable	119/400 (29.8)	117/390 (30.0)	1.00 (0.92 to 1.10)	0.939
Unfavourable	281/400 (70.3)	273/390 (70.0)	0.2 (-6.1 to 6.6)	0.939

Table 8: Neurological outcome restricted to those surviving at 3 months

Neurological Outcome				
	Cardiac arrest centre n=414	Standard care n=413	Risk ratio	P value
Modified Rankin Score at discharge				
0	68/144 (47.2)	75/137 (54.7)		
1	21/144 (14.6)	31/137 (22.6)		
2	22/144 (15.3)	12/137 (8.8)		
3	13/144 (9.0)	8/137 (5.8)		
4	9/144 (6.3)	2/137 (1.5)		
5	11/144 (7.6)	9/137 (6.6)	1.55 (1.00 to 2.41)	0.050
Modified Rankin Score at 3 months				
0	69/127 (54.3)	75/132 (56.8)		
1	32/127 (25.2)	22/132 (16.7)		
2	9/127 (7.1)	17/132 (12.9)		
3	9/127 (7.1)	5/132 (3.8)		
4	3/127 (2.4)	9/132 (6.8)		
5	5/127 (3.9)	4/132 (3.0)	1.01 (0.63 to 1.61)	0.965
Cerebral Performance Category				
1	98/143 (68.5)	106/137 (77.4)		
2	23/143 (16.1)	18/137 (13.1)		
3	15/143 (10.5)	9/137 (6.6)		

4	7/143 (4.9)	4/137 (2.9)	1.59 (0.94 to 2.70)	0.084

Table 9: EQ-5D-5L at discharge

	Cardiac Arrest Centre	Standard care	Difference in means (95% CI)	p-value
Mobility				
No problems	61/97 (62.9)	62/92 (67.4)		
Slight problems	18/97 (18.6)	16/92 (17.4)		
Moderate problems	7/97 (7.2)	12/92 (13.0)		
Severe problems	6/97 (6.2)	0/92 (0.0)		
Unable to walk about	5/97 (5.2)	2/92 (2.2)		
Self-care				
No problems	58/97 (59.8)	60/92 (65.2)		
Slight problems	16/97 (16.5)	22/92 (23.9)		
Moderate problems	11/97 (11.3)	6/92 (6.5)		
Severe problems	8/97 (8.2)	2/92 (2.2)		
Unable to wash or dress	4/97 (4.1)	2/92 (2.2)		
Usual activities				
No problems	38/97 (39.2)	41/92 (44.6)		
Slight problems	20/97 (20.6)	23/92 (25.0)		
Moderate problems	15/97 (15.5)	14/92 (15.2)		
Severe problems	10/97 (10.3)	5/92 (5.4)		

Unable to do usual activities	14/97 (14.4)	9/92 (9.8)		
Pain/discomfort				
No pain/discomfort	44/97 (45.4)	32/92 (34.8)		
Slight pain/discomfort	27/97 (27.8)	38/92 (41.3)		
Moderate pain/discomfort	20/97 (20.6)	18/92 (19.6)		
Severe pain/discomfort	6/97 (6.2)	2/92 (2.2)		
Extreme pain/discomfort	0/97 (0.0)	2/92 (2.2)		
Anxiety/depression				
Not anxious/depressed	53/97 (54.6)	55/92 (59.8)		
Slightly anxious/depressed	25/97 (25.8)	18/92 (19.6)		
Moderately anxious/depressed	15/97 (15.5)	15/92 (16.3)		
Severely anxious/depressed	4/97 (4.1)	2/92 (2.2)		
Extremely anxious/depressed	0/97 (0.0)	2/92 (2.2)		
EQ-5D-5L index, mean (SD)	0.68 (0.32), n=97	0.72 (0.25), n=92	-0.04 (-0.12 to 0.05)	0.379

EQ-5D-5L visual analogue scale, mean (SD)	66.3 (22.2), n=95	69.8 (19.3), n=88	-3.50 (-9.59 to 2.59)	0.259

Table 10: Subgroup analyses for all-cause mortality at 30 days (ITT population)

	Cardiac Arrest Centre	Standard care	Risk ratio (95% CI)	Interaction p-value
Age (years)				
<57	53/121 (43.8)	87/151 (57.6)	0.76 (0.60 to 0.97)	
57 to 71	103/153 (67.3)	70/133 (52.6)	1.28 (1.05 to 1.56)	
≥72	100/135 (74.1)	101/128 (78.9)	0.94 (0.82 to 1.07)	0.003
Gender				
Female	92/127 (72.4)	98/135 (72.6)	1.00 (0.86 to 1.16)	
Male	164/282 (58.2)	157/274 (57.3)	1.01 (0.88 to 1.17)	0.872
Presenting cardiac rhythm*				
AED non-shockable/asystole/PEA	167/184 (90.8)	173/188 (92.0)	0.99 (0.93 to 1.05)	
AED shockable/VF/pulseless VT	90/226 (39.8)	85/224 (37.9)	1.05 (0.83 to 1.32)	0.455
Witnessed arrest*				
No	64/76 (84.2)	71/81 (87.7)	0.96 (0.85 to 1.09)	
Yes	194/335 (57.9)	187/331 (56.5)	1.03 (0.90 to 1.17)	0.435
Bystander CPR				
No	92/123 (74.8)	74/99 (74.7)	1.00 (0.86 to 1.17)	

Yes	166/288 (57.6)	184/312 (59.0)	0.98 (0.85 to 1.12)	0.822
Location of arrest				
Private	161/208 (77.4)	179/242 (74.0)	1.05 (0.94 to 1.16)	
Public	97/203 (47.8)	79/170 (46.5)	1.03 (0.83 to 1.28)	0.886
COVID-19				
Randomised pre 11th March 2020	187/282 (66.3)	183/282 (64.9)	1.02 (0.91 to 1.15)	
Randomised post 11th March 2020	71/129 (55.0)	75/130 (57.7)	0.95 (0.77 to 1.18)	0.583
Time to ROSC (mins)**				
<25	50/154 (32.5)	46/156 (29.5)	1.04 (0.66 to 1.64)	
≥25	136/154 (88.3)	137/157 (87.3)	1.00 (0.53 to 1.87)	0.915
Age (years)***				
<50	26/68 (38.2)	41/78 (52.6)	0.73 (0.50 to 1.05)	
50 to <60	37/71 (52.1)	57/93 (61.3)	0.85 (0.65 to 1.12)	
60 to <70	80/117 (68.4)	51/98 (52.0)	1.31 (1.05 to 1.65)	
70 to <80	65/93 (69.9)	55/78 (70.5)	0.99 (0.81 to 1.21)	
≥80	48/60 (80.0)	54/65 (83.1)	0.96 (0.81 to 1.14)	0.041

* due to convergence issues, interaction p-values estimated using Mantel-Haenszel tests.

** following multiple imputation of missing values. Due to convergence issues, odds ratios and interaction p-value estimated using logistic regression.

*** post hoc analysis

Table 11: Adverse events in the ITT population

	Cardiac Arrest Centre	Standard care
	(n=414)	(n=413)
Number of patients with an adverse event		
Serious adverse events (SAE)	8/414 (1.9)	3/413 (0.7)
Non-serious adverse events (NSAE)	2/414 (0.5)	1/413 (0.2)
Total number of adverse events		
Serious adverse events (SAE)	8	5
Non-serious adverse events (NSAE)	3	1

All SAEs and NSAEs were deemed unrelated to the trial interventions by the “investigator”

Study Title: A randomised trial of expedited transfer to a cardiac arrest centre for non-ST elevation out-of-hospital cardiac arrest (ARREST)

Study Registration Number: ISRCTN96585404

SAP Author: Matt Dodd, Mia Tackney and Tim Clayton

SAP Author (Health Economic Analysis): Paul McCrone

SAP Version: 1.0

SAP date: 28th April 2023

FINAL VERSION APPROVED BY: Simon Redwood, Tim Clayton

1 Introduction....4
2 Study Synopsis 4
3 Study Objectives 5
3.1 Primary objective 5
3.2 Secondary objectives....5
3.3 Changes to the primary objective during the conduct of the study....5
4 Study Design 5
4.1 General design and plan....5
4.2 Sample size....5
4.3 Randomisation and blinding 6
4.4 Study assessments....7
4.5 Adjudication of outcomes....7
5 Study Populations....7
5.1 Subject disposition 7
5.2 Definition of populations for analysis 8
5.3 Intent-to-treat....8
5.4 Per protocol....8
5.5 As-treated populations....8
5.6 Major protocol deviations....9
5.7 Definition of subgroup population in different analyses 9
6 Statistical Analysis....9
6.1 General 9
6.2 Pooling of sites 10
6.3 Interim analyses 10
6.4 Time-points for analysis 10
6.5 Methods for handling withdrawals and missing data....10
6.5.1 Withdrawals....10
6.5.2 Missing data....10

6.6 Statistical analytical issues..10
7 Evaluation of Demographics, Baseline Characteristics 11
7.1 Demographics and baseline characteristics...11
7.2 Medical history..11
7.3 In-hospital care 12
7.4 Prior therapies and medications...13
8 Evaluation of Treatment Compliance...13
8.1 Compliance with study intervention..13
9 Evaluation of Efficacy parameters...14
9.1 Analysis of primary endpoint – all cause mortality 30 days after randomisation.........14
9.2 Analysis of secondary, and other efficacy endpoints 14
9.2.1 All-cause mortality at 3, 6, and 12 months..14
9.2.2 Neurological status...14
9.2.3 EQ-5D-5L at discharge..15
9.3 Impact of COVID-19 16
10 Evaluation of Safety Parameters..16
10.1 Adverse events (AEs)..16
11 Analysis of Health Economic Outcomes...16
12 References 18
Appendix: Figures 19
Figure 1: Trial flow diagram ARREST 19
Figure 2: Trial procedures table 20

1 Introduction

This Statistical Analysis Plan (SAP) describes the planned analysis and reporting for the clinical trial entitled: A randomised trial of expedited transfer to a cardiac arrest centre (CAC) for non-ST elevation out-of-hospital cardiac arrest (ARREST).

The following documents were reviewed in preparation of this charter:

- ARREST trial protocol version 6, issued 17th July 2019
- Site case report form (CRF) version 2, issued 21st June 2019
- London Ambulance Service (LAS) CRF version 2, issued 21st June 2019

The reader of this SAP is encouraged to also read the ARREST protocol for details on the conduct of this study, and the operational aspects of clinical assessments and timing for completing a patient in this study.

2 Study Synopsis

ARREST is a randomised controlled intervention trial. It is being undertaken to determine the best post-resuscitation care pathway for patients without ST-segment elevation (STE). Specifically, it proposes that changes to emergency management comprising expedited delivery to a CAC will reduce mortality in patients without STE compared to the current standard of care.

Patients will be randomised with 1:1 ratio to either the control (current standard of care) or intervention arm (expedited transfer to a CAC). In total 430 patients will be randomised to the intervention arm and 430 patients will be randomised to the control arm (Figure 1).

The intervention arm consists of activation of the pre-hospital triaging system currently in place for post-arrest STE patients. This involves pre-alert of the CAC and strategic delivery of the patient to the catheter laboratory (24 hours a day, 7 days a week). Patients will receive definitive post-resuscitation care: intubation and ventilation, where necessary, targeted temperature management, and goal-directed therapies including evaluation and identification of underlying cause of arrest with access to immediate reperfusion if necessary. Prognostication will occur no earlier than 72 hours post-cardiac arrest to prevent premature withdrawal of life-sustaining treatment. Transfer times estimated from the 40-patient pilot are anticipated to be 100 minutes (median; IQR 75 to 113) from time of arrest to the designated centre.

The control arm comprises the current standard of pre-hospital advanced life support (ALS) care management for patients with return of spontaneous circulation (ROSC) following cardiac arrest of suspected cardiac aetiology. The patient is conveyed to the geographically closest emergency department. Management thereafter will be as per standard hospital protocols however as in the intervention arm, prognostication is to be delayed in trial patients until at least 72 hours post arrest.

3 STUDY OBJECTIVES

3.1 Primary objective

To determine the effect of expedited transfer to a CAC on all-cause mortality at 30 days in patients who experience out-of-hospital cardiac arrest (OHCA).

3.2 Secondary objectives

To determine the effects of expedited transfer to a CAC on:

- Neurological status at discharge and 3 months
- All-cause mortality at 3, 6 and 12 months
- EQ-5D-5L at discharge
- Health care costs and cost-effectiveness

3.3 Changes to the primary objective during the conduct of the study

No changes to the primary objective have been made during the conduct of the study.

4 STUDY DESIGN

4.1 General design and plan

A randomised trial of expedited transfer to a CAC for non-ST elevation out-of-hospital cardiac arrest. This is a London-based trial with participation by the LAS, seven CAC sites, and 34 emergency sites. There are two trial arms: the control arm (current standard of care) and the intervention arm (expedited transfer to a CAC).

4.2 Sample size

The primary endpoint will be all-cause mortality at 30 days in patients who experience OHCA. Mortality at 30 days in the control arm is expected to be approximately 60% for the type of patients recruited into ARREST, based on Pan London OHCA data (87% mortality with ROSC at any time post cardiac arrest and 73% mortality with ROSC

maintained to hospital), registry data and the pilot study.

Observational studies on implementation of treatment bundles have shown absolute risk reductions (ARR) of near 30% compared to the baseline comparator and the Parisian group have shown ARR of 16% following PCI in non-STE. If half of the population in question will have a treatable lesion and the combined treatment effect of this within a treatment bundle is examined, a 10% ARR will be practical from 60% to 50% mortality.

A trial of 860 patients (430 in each arm) provides 80% to detect an absolute reduction of 10% (from 60% to 50%) with up to 10% losses to follow-up and a 5% significance level. If the mortality rate is higher than 60% then the power will increase to detect a 10% absolute reduction in mortality.

4.3 Randomisation and blinding

Designated LAS staff at the Advanced Paramedic Practitioner (APP) dispatch desk will randomise patients into the intervention arm or control arm using the following procedure:

- Paramedics attending a suspected OHCA will assess the patient for eligibility. Once eligibility is confirmed, the on-site paramedic will ask the APP desk to randomise the patient into the trial.
- The on-site paramedics will call the APP dispatch desk to provide the patient details required to complete randomisation.
- The APP desk staff will access the randomisation site, enter the required details, and generate a study ID and treatment allocation. Patients will be randomised either to an expedited transfer to a CAC or to receive standard of care.
- The APP desk will inform the on-site paramedic which group the patient has been randomised to, and the on-site paramedic will proceed as appropriate.
- The APP desk will provide the necessary information to the Clinical Audit and Research Unit (CARU) at LAS for them to track the patient report form (PRF) to gain clinical data.

Access to the randomisation site www.sealedenvelope.com will be strictly controlled and available only to delegated staff of the APP desk at LAS that have received appropriate training.

At the LSHTM clinical trials unit only the unblinded trial statistician will have access to the randomisation codes. There is no attempt to blind participants, investigators or follow-up staff given the nature of the intervention.

4.4 Study assessments

Study assessments are detailed in Figure 2. All patients have a ROSC assessment pre-hospital and on arrival to the hospital, and mortality status is recorded at the hospital. The patients will be followed up at 30 days, 3 months, 6 months and 12 months after the cardiac arrest, in order to determine outcomes contributing to the primary and secondary endpoints. Delegated research nurses will collect data for in-hospital care and the 30-day and 3-month follow up time points. Delegated research nurses at St Thomas' will collect mortality data for the 6-month and 12-month follow-up time points.

4.5 Adjudication of outcomes

There is no adjudication of all-cause mortality or any other outcomes.

5 STUDY POPULATIONS

Patients with confirmed cardiac arrest will be assessed for eligibility by the attending LAS paramedic. Due to the emergency context of the research, identification cannot be performed in advance. The frequency and percent of subjects in each analysis population, study withdrawals, and major protocol violations will also be presented in the form of a CONSORT diagram.

5.1 Subject disposition

Inclusion criteria:

1. Out-of-hospital cardiac arrest (OHCA)
2. Return of spontaneous circulation (ROSC)
3. Age 18 or over (known or presumed)

Exclusion criteria:

1. Criteria for ST-elevation myocardial infarction on 12-Lead electrocardiogram (ECG)
2. Do Not Attempt Resuscitation (DNAR) Order
3. Cardiac arrest suffered after care pathway set and patient en route
4. Suspected pregnancy

5. Presumed non-cardiac cause (for example; trauma, drowning, suicide, drug overdose)
6. Presumed significant trauma/injury

5.2 Definition of populations for analysis

The primary analysis will be performed on an intention to treat basis, by including all patients where possible according to the group to which they were randomised regardless of their adherence with the entry criteria, treatment actually received, and subsequent withdrawal from treatment or deviation from the protocol. A secondary per protocol analysis will be undertaken including only patients who receive the allocated intervention as intended. Further secondary analyses will be undertaken according to where patients were admitted detailed below.

5.3 Intent-to-treat

The intent-to-treat analysis population will include all patients randomised to ARREST regardless of whether they experienced their randomised intervention as specified (i.e. including all patients whether or not they underwent expediated transfer to a CAC or to an emergency department as indicated by the treatment allocation). This will be the focus for the primary results paper with results from any other analysis population presented in the Supplementary Material unless specifically requested by the relevant journal.

5.4 Per protocol

The per protocol analysis population will include patients randomised to ARREST who experienced their randomised intervention as specified. Patients who crossover to the alternative pathway will be excluded.

5.5 As-treated populations

To assess whether any impact of treatment arises from admission to a CAC irrespective of whether this is to the emergency department or to the cardiac catheter laboratory two further analyses will be undertaken according to where the patient was admitted.

The first analysis will compare all those who go direct to a CAC (whether to the emergency department or cardiac catheter laboratory) with those who go to a district general hospital.

The second analysis will consider patients in three groups (i) those who go directly to a cardiac catheter laboratory, (ii) those who go the emergency department in a CAC, and (iii) those who go to the emergency department in a district general hospital. For this analysis the main comparisons will be with those who go to the district general hospital (ie. group (i) vs. group (iii) and group (ii) vs. group (iii)).

5.6 Major protocol deviations

It is not anticipated that there will be protocol violations and deviations that would affect the statistical analysis although these will be fully described.

5.7 Definition of subgroup population in different analyses

A limited number of subgroup analyses on the primary endpoint are planned: these include patients randomised pre/post 11th March 2020 when recruitment was paused due to COVID-19 (see section 9.3), gender, age group (in tertiles), initial shockable rhythm, whether the cardiac arrest was witnessed or not, whether bystander CPR was administer, the duration of the cardiac arrest until ROSC (using the median time as the cutpoint), and location of cardiac arrest (at home or in a public place). These analyses involve generalised linear models (GLMs) for a binomial outcome (log link function) including an interaction between the characteristic and the intervention with effect estimates and confidence intervals produced. In addition, a subgroup analysis will be undertaken by developing a model using GLMs for a binomial outcome, categorising patients according to their underlying risk of dying within 30 days. This simultaneously accounts for the multiple risk factors a patient may have at baseline and will assess whether intervention is more effective at higher underlying risk again using interactions tests. Depending on the level of missing data for time to ROSC a separate category will be incorporated for those with a missing time to ROSC and additional analyses will utilise multiple imputation techniques.

6 Statistical Analysis

6.1 General

The final statistical analysis will be performed as pre-specified in this SAP. Any, post-hoc, exploratory analyses completed to support planned analyses, which were not identified in this SAP, will be documented and reported in the relevant trial reports. Any results from unplanned analyses will be clearly identified in the text of the trial reports.

6.2 Pooling of sites

The data from each participating CAC site and emergency department will be pooled for all primary and secondary analyses.

6.3 Interim analyses

A Data and Safety Monitoring Committee (DSMC) was established to monitor safety and efficacy. This was the only group, along with the statistician producing the reports for the DSMC, to see interim analyses by treatment. There were no formal stopping rules for efficacy or futility based on observed treatment differences in this study.

6.4 Time-points for analysis

The analyses will be undertaken at two timepoints (i) 3 months (when the key outcomes at 30 days, discharge and 3 months will be available), and (ii) 12 months after the last patient has been randomised. No analyses identified in the protocol and in this SAP will be performed until after the last patient has completed their 30-day follow-up. In addition, no database may be locked, randomisation code unblinded, or analyses completed until this SAP has been approved other than for any specified interim analyses.

6.5 Methods for handling withdrawals and missing data

6.5.1 Withdrawals

A patient may decide to withdraw from the trial at any time without prejudice to their future care. If the patient has previously consented, NHS records will continue to be used to gather endpoint data unless the patient explicitly denies permission for us to do this.

6.5.2 Missing data

Missing data will be identified and an effort made to return to the original medical records to obtain the data. If this is not possible and the data are missing multiple imputation methods will be used to impute values in the baseline variables used for and adjusted analyses. Large amounts of missing outcome data are not expected. However, the analyses involving missing CPC and mRS scores and EQ-5D-5L are detailed in sections 9.2.2 and 9.2.3.

6.6 Statistical analytical issues

Where a patient dies without providing written informed consent, the following times to death will be imputed:

- <48 hours = 1 day
- 48 hours to 7 days = 4.5 days
- 8 days to 14 days = 11 days
- 15 days to 30 days = 22.5 days
- 31 days to 3 months = 61 days
- 3 months to 6 months = 137 days
- 6 months to 12 months = 274 days

These imputed times to event correspond with the midpoints of the ranges provided.

Data analysis will be performed with Stata® Version 17.0 or later. Other statistical software may also be used where Stata® does not provide the relevant statistical method.

7 EVALUATION OF DEMOGRAPHICS, BASELINE CHARACTERISTICS

For all patient characteristics, continuous variables will be described by the mean and standard deviation except for skewed variables which may be described by the median and inter quartile range (IQR). Categorical variables will be described by frequency and percentages in each category.

7.1 Demographics and baseline characteristics

The following demographic characteristics are collected:

- Age
- Gender
- Ethnicity

7.2 Medical history

The following information will be collected on medical history:

- Diabetes
- Hypertension
- Smoking Status
- Hypercholesterolaemia
- Cholesterol level on admission (if taken)
- Peripheral vascular disease
- Cerebrovascular disease
- Chronic renal failure
- Known ischaemic heart disease

- Previous MI
- Previous PCI
- Family history of CV disease
- Preceding symptoms prior to arrest
- Cerebral performance category (CPC) score on arrival to the hospital
- Modified Rankin Scale (mRS) score on arrival to the hospital

7.3 In-hospital care

- Arrest-to-door time
- Cardiogenic shock
- Arterial blood gas on admission, if yes, pH
- Angiogram attempted
    - Extent of disease
    - Number of diseased vessels
- TIMI flow in Culprit vessel (pre-PCI)
- Revascularisation
    - If PCI, which artery was intervened on
    - Total number of vessels treated
    - PCI success
- TIMI flow in Culprit vessel (post-PCI)
- Circulatory support
- Renal support
- Ventilatory support
- Targeted temperature management
- Troponin (T or I)
- Admission creatinine, if measured
    - Creatinine value
- LV function
    - Level if assessed on admission
    - Level if assessed prior to discharge
    - ICD insertion
- Reinfarction
- Unplanned revascularisation of Target Vessel or Lesion
- Stroke (does not include anoxic brain injury)
- If yes, embolic or haemorrhagic

- Sepsis
- Bleeding up to discharge
    - If yes, type
- Unexpected adverse events

7.4 Prior therapies and medications

The following information will be collected on medication use prior to admission:

- Aspirin
- Additional antiplatelet medication
- β-blocker
- Warfarin
- Other anticoagulant
- ACE inhibitor
- Angiotensin receptor blocker
- Aldosterone antagonist
- Statin
- Amiodarone
- Digoxin
- Loop or Thiazide Diuretics
- Insulin
- Oral hypoglycaemic agent
- Other

8 Evaluation of Treatment Compliance

8.1 Compliance with study intervention

Information will be collected on whether the patient underwent expedited transfer to a CAC or to an emergency department as indicated by their treatment allocation and whether care was completed as planned. This will be presented as part of the study report. Analyses will be undertaken according to the intervention received (see section 5).

9 EVALUATION OF EFFICACY PARAMETERS

9.1 Analysis of primary endpoint – all cause mortality 30 days after randomisation.

The primary analysis will be a comparison of all-cause mortality 30 days after randomisation between the two arms. A risk ratio together with a 95% confidence interval and p-value will be calculated using a GLM for binomial outcomes with a log link function. The risk difference in all-cause mortality at 30 days will be calculated using a GLM for binomial outcomes using the identity link function. For the as-treated analysis with patients considered in three groups the above GLM model will be fitted but with the intervention entered as a three-group co-variate with the group admitted to the emergency department in a district general hospital as the reference group. The primary analysis will be unadjusted with a secondary analysis undertaken adjusting for those variables detailed in section 5.7 with age and duration of arrest until ROSC entered as continuous variables with appropriate transformation and use of multiple imputation techniques if necessary. If a risk ratio model is not able to be fitted a logistic regression model will be used for the adjusted model.

9.2 Analysis of secondary, and other efficacy endpoints

9.2.1 All-cause mortality at 3, 6, and 12 months

Similarly to the primary endpoint, a risk ratio together with a 95% confidence intervals and a p-value will be calculated together with the risk difference for all-cause mortality at 3, 6 and 12 months. Kaplan-Meier curves will be produced to show all-cause mortality up to 12 months. Hazard ratios and corresponding 95% confidence intervals and p-values will be calculated using Cox proportional hazards models. The proportional hazards assumption will be assessed visually using Nelson-Aalen graphs and by formally testing the Schoenfeld residuals.

9.2.2 Neurological status

Neurological status at discharge and 3 months will be compared using the mRS score (an ordinal score from 0 (no symptoms) to 6 (dead). This will be the primary outcome measure of neurological status in accordance with recent international guidelines.[1] However, the CPC score (an ordinal score from 1 (normal neurological status) to 5 (dead)) was also measured since this is commonly used in assessing neurological status post-cardiac arrest and will also be compared. Ordered logistic regression will be used to compare the two treatments. The effect of treatment will be estimated

using proportional odds ratios from mixed effects ordinal logistic regression models containing indicators for treatment, visit (discharge and 3 months) and their interaction. We will also undertake two sensitivity analyses, both adjusting for baseline values of the respective outcome, to assess the impact of missing values: (i) compare observed scores at each time point separately using standard ordinal logistic regression, and (ii) compare observed scores at each timepoint imputing the last observation carried forward for missing values. If there is clear evidence against the proportional odds assumption, comparisons will also be made by dichotomising the outcome or using appropriate non-parametric methods.

Completion of the mRS and CPC was challenging and as a result there are missing data likely for these scores. In addition, while mRS collection was introduced early in the trial, the first patients only had CPC score collected. Centres have been requested to complete as much information which may be limited to a favourable/non-favourable score for mRS and/or CPC. Therefore three further analyses will be undertaken to maximise the data available.

The first two analyses will compare mRS and CPC between interventions as binary variables with risk ratios and risk differences together with 95% confidence intervals. For mRS the threshold will be 0-3 (favourable) and 4-6 (unfavourable), and for CPC the cutpoint will be 1-2 (favourable) and 3-5 (unfavourable). Since some patients will only have one score recorded the third analysis will use a favourable outcome on either score as the outcome and again risk ratio and risk differences presented.

Given the expected level of mortality a sensitivity analysis will be undertaken repeating the above analyses among survivors only.

9.2.3 EQ-5D-5L at discharge

Scores on the EQ-5D-5L questionnaire at discharge will be converted into a quality of life index value using the most appropriate UK specific value set available at the time of analysis. Differences in the mean EQ-5D-5L index value together with 95% confidence intervals will be calculated using linear regression models. The distribution of the outcome variable will be investigated for non-normality and if necessary a data transformation will be made or a non-parametric statistical analysis will be conducted. Longer term outcomes to 12 months will be analysed using linear mixed models for repeated measures. The above analyses will also be undertaken for the EQ-5D-5L Visual Analogue Scale. Components of the EQ-5D-5L will also be reported.

9.3 Impact of COVID-19

Recruitment was paused on 11th March 2020 due to the impact of the COVID-19 pandemic. To allow for potential increase in event rates due to the COVID-19 pandemic and the additional associated challenges additional analyses will be undertaken.

The event rates for all-cause death will be compared pre- and post- 11th March 2020 when recruitment was paused. An analysis will be undertaken of the primary and secondary outcomes comparing the impact of the interventions pre- and post- 11th March 2020. The intervention effect together with 95% confidence intervals will presented by these time periods and formally assessed with an interaction test between time period and treatment from the Cox model.

10 Evaluation of Safety Parameters

10.1 Adverse events (AEs)

AEs will be reported as the proportion in each study intervention group that suffer an AE.

A data listing of serious adverse events (SAEs) will be provided, displaying details of the event(s) captured on the SAE form. This listing will include those patients who rearrest en route to the hospital.

A data listing of deaths will be supplied, displaying details of the cause of death.

11 Analysis of Health Economic Outcomes

Inpatient and outpatient use is at 30 days and 3 months follow-up. This information will be converted into costs using NHS Reference Cost data. Other data informing the economic analyses will be taken from hospital records on procedures where these are available. Costs of in-hospital care will be also be calculated along with journey times. Costs at 30 days and 3 months will be compared between the groups using bootstrapped models due to the expected positive skewness in the data.

Cost-effectiveness will be assessed by combining costs with the primary outcome and with quality-adjusted life years (QALYs) at 30 days derived from the EQ-5D-5L. Given it is not possible to record the EQ-5D-5L at the point of randomisation it will be assumed that the health state 'unconsciousness' will apply and sensitivity analyses will be conducted by varying this value. QALYs are calculated using area under the curve

methods which typically assumes a linear change between time points. Alternative rates of change will be used in sensitivity analyses.

Incremental cost-effectiveness ratios (difference in costs divided by difference in outcomes) will be calculated for mortality and QALYs. Uncertainty will be examined using cost-effectiveness planes derived from 1000 bootstrapped resamples and cost-effectiveness acceptability curves. Missing data for individual service items will be replaced with median values from valid cases. Missing total costs and QALYs will be replaced via multiple imputation.

12 References

1. Haywood K, Whitehead L, Nadkarni et al. COSCA (Core Outcome Set for Cardiac Arrest) in Adults An Advisory Statement From the International Liaison Committee on Resuscitation. Circulation. 2018;137:e783–e801

Appendix: Figures

Figure 1: Trial flow diagram ARREST

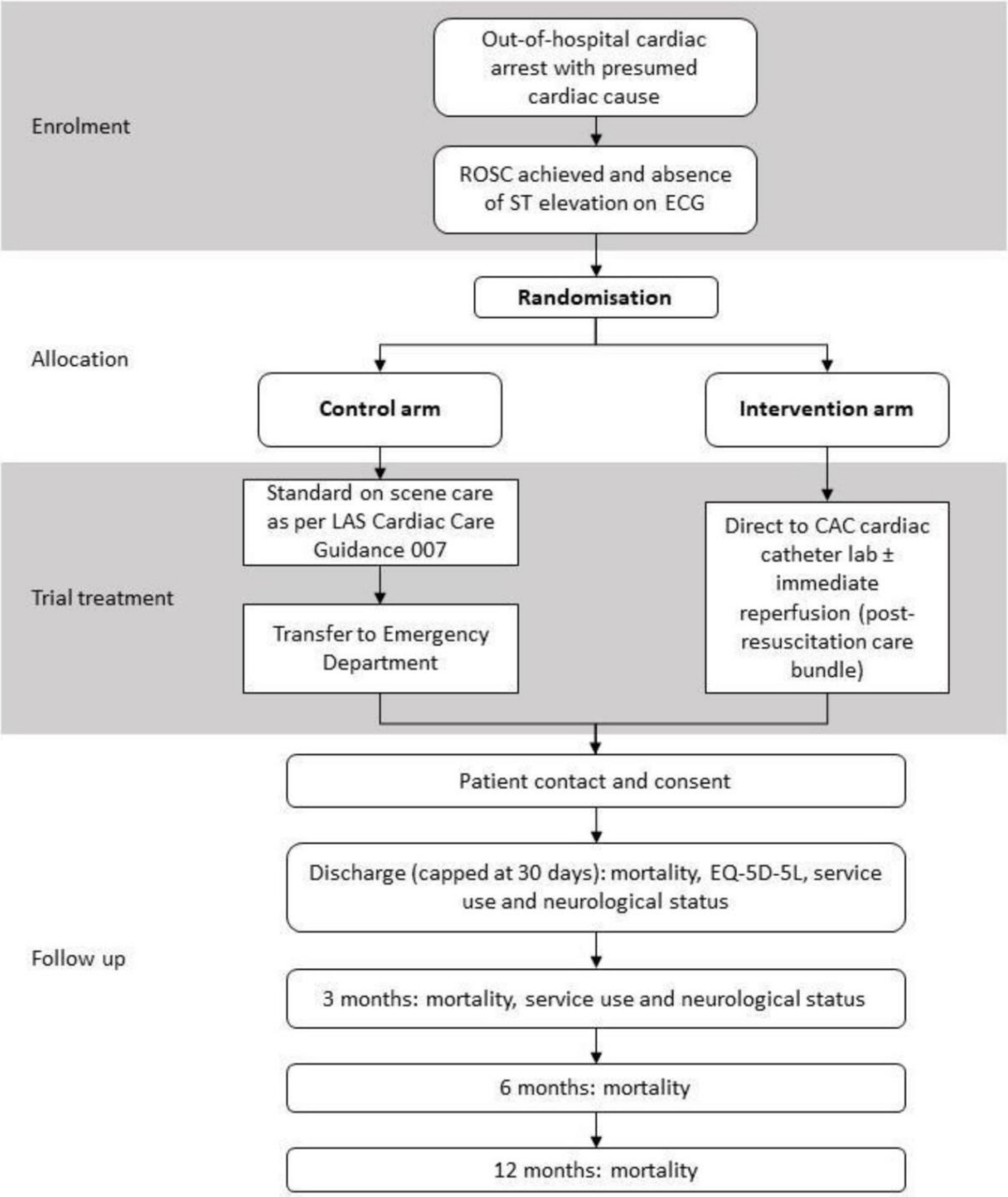

Figure 2: Trial procedures table

	Pre-hospital	On arrival to hospital	In hospital	Discharge (capped at 30 days)	3 months	6 months	12 months
Review of eligibility criteria	X						
ROSC assessment	X	X					
Randomisation	X						
Transfer to CAC or hospital	X						
PIS & Informed consent / Personal Consultee / Professional Consultee			X				
Neurological status			X		X		
Mortality status		X	X	X	X	X	X
EQ-5D-5L				X			
Service use questionnaire				X	X		
SAE / NSAEs	X	X	X	X	X		
